# Comprehensive ethological analysis of fear expression in rats using DeepLabCut and SimBA machine learning model

**DOI:** 10.3389/fnbeh.2024.1440601

**Published:** 2024-08-01

**Authors:** Kanat Chanthongdee, Yerko Fuentealba, Thor Wahlestedt, Lou Foulhac, Tetiana Kardash, Andrea Coppola, Markus Heilig, Estelle Barbier

**Affiliations:** ^1^Department of Biomedical and Clinical Sciences, Center for Social and Affective Neuroscience, Linköping University, Linköping, Sweden; ^2^Department of Physiology, Faculty of Medicine Siriraj Hospital, Mahidol University, Bangkok, Thailand; ^3^Bordeaux Neurocampus, University of Bordeaux, Bordeaux, France

**Keywords:** fear conditioning, ethological analysis, risk-assessment, DeepLabCut, SimBA

## Abstract

**Introduction:**

Defensive responses to threat-associated cues are commonly evaluated using conditioned freezing or suppression of operant responding. However, rats display a broad range of behaviors and shift their defensive behaviors based on immediacy of threats and context. This study aimed to systematically quantify the defensive behaviors that are triggered in response to threat-associated cues and assess whether they can accurately be identified using DeepLabCut in conjunction with SimBA.

**Methods:**

We evaluated behavioral responses to fear using the auditory fear conditioning paradigm. Observable behaviors triggered by threat-associated cues were manually scored using Ethovision XT. Subsequently, we investigated the effects of diazepam (0, 0.3, or 1 mg/kg), administered intraperitoneally before fear memory testing, to assess its anxiolytic impact on these behaviors. We then developed a DeepLabCut + SimBA workflow for ethological analysis employing a series of machine learning models. The accuracy of behavior classifications generated by this pipeline was evaluated by comparing its output scores to the manually annotated scores.

**Results:**

Our findings show that, besides conditioned suppression and freezing, rats exhibit heightened risk assessment behaviors, including sniffing, rearing, free-air whisking, and head scanning. We observed that diazepam dose-dependently mitigates these risk-assessment behaviors in both sexes, suggesting a good predictive validity of our readouts. With adequate amount of training data (approximately > 30,000 frames containing such behavior), DeepLabCut + SimBA workflow yields high accuracy with a reasonable transferability to classify well-represented behaviors in a different experimental condition. We also found that maintaining the same condition between training and evaluation data sets is recommended while developing DeepLabCut + SimBA workflow to achieve the highest accuracy.

**Discussion:**

Our findings suggest that an ethological analysis can be used to assess fear learning. With the application of DeepLabCut and SimBA, this approach provides an alternative method to decode ongoing defensive behaviors in both male and female rats for further investigation of fear-related neurobiological underpinnings.

## 1 Introduction

Fear is an aversive emotion that is essential for surviving threats to both physical and psychological well-being (Steimer, 2002). However, excessive, or maladaptive fear can lead to the development of psychiatric disorders such as anxiety disorders and post-traumatic stress disorder (PTSD) (American Psychiatric Association, 2013; Craske et al., 2017). This has prompted the necessity for a deeper understanding of the mechanisms underlying fear responses and their regulation. While fears can be innate, they can also be acquired through learning. This acquisition often occurs through the association of a threat with environmental cues, allowing its exploration through Pavlovian conditioning.

Exposure to threats or threat-associated cues elicits a shift in behavior, characterized by a decrease in appetitive behaviors (e.g., food procurement and sexual behaviors) and an increase in defensive behaviors. The assessment of decreased appetitive behaviors in response to threats utilizes the conditioned suppression paradigm pioneered by Estes and Skinner (1941). In conditioned suppression, the presentation of a cue previously associated with threatening events suppresses consummatory behavior quantified as operant responding. The strength of this approach lies in its objective and easily quantified measurement of an operant behavior, and its pharmacological validation by anxiolytic drugs such as benzodiazepines. Defensive behaviors triggered by threat-associated cues can also be evaluated by measuring freezing behavior, a natural defensive response in rodents that is detectable in a laboratory setting. The latter approach has been the most common readout in recent years and has uncovered crucial information regarding the neurobiological mechanisms of fear learning (Maren and Fanselow, 1996; LeDoux, 2000).

However, neither conditioned suppression nor freezing are the sole behavioral responses that can be triggered by a threat. In rats, the selection of defensive behaviors is dynamic and influenced by updated information regarding the immediacy of threats (Fanselow and Lester, 1988; Blanchard and Blanchard, 1989b; Moscarello and Penzo, 2022). For instance, rats are more likely to flee to avoid confrontation in escapable situations (Blanchard and Blanchard, 1971) whereas they tend to hide or freeze when threats are distant. In situations with ambiguous threat immediacy, rats tend to gather more sensory information through behaviors like head-scanning, sniffing, rearing, and stretched-approaching, collectively termed as “vigilance” or “risk assessment” (Blanchard and Blanchard, 1989a; Misslin, 2003).

According to Blanchard and colleagues, risk assessment does not only help optimize defensive choices in the face of threats but also facilitates a return to non-defensive behaviors afterwards (Blanchard et al., 2011). Fanselow and Lester proposed that these defensive behaviors are organized along a predatory imminence continuum, where physical closeness, temporal immediacy, and the likelihood of encountering the threat will determine the behavioral response (Fanselow and Lester, 1988). Following this theory, the defensive behavior system can be divided into three modes that are activated by different levels of fear, including pre-encounter (vigilance, risk assessment), post-encounter (freezing), and circa-strike defensive behaviors. Failure in selecting the appropriate mode of defense may reflect maladaptive behavioral responses. For instance, abnormalities in risk assessment can lead to prolonged defensive states and delay the return to normalcy, as observed in cases of excessive fear, anxiety, or the hypervigilance of PTSD (Shumake and Monfils, 2015; Beckers et al., 2023). Thus, a comprehensive ethological analysis of rat behaviors elicited by threat-associated stimuli could enhance our understanding of their responses to threats and provide insights into the underlying mechanisms.

Despite the complexity of fear responses outlined above, these have predominantly been assessed through simpler, more easily quantifiable measurements, such as suppression of operant responding (i.e., conditioned suppression) and freezing behaviors. This is largely because accurately measuring multiple defensive behaviors poses substantial challenges due to the limited sensitivity of commercial tracking systems (Sturman et al., 2020). As a result, human annotation has remained the gold standard method for quantifying ethological behaviors, despite being time-consuming and prone to subjective bias. Recently, advances in computer vision and machine learning have provided new tools poised to attain human-level accuracy and establish standardized behavioral scoring. For instance, the open-source toolbox DeepLabCut allows behavioral tracking by extracting the poses of animals without using markers (Mathis et al., 2018). It relies on deep learning using neural networks to establish pose estimation data for a variety of animals. Available studies report a high level of accuracy with a relatively low requirement for training data, making it an attractive tool for measuring ethological behaviors (Hardin and Schlupp, 2022). DeepLabCut can be used in combination with SimBA (Simple Behavioral Analysis) which integrates pose estimation data from DeepLabCut with human-labeled behavioral annotations to autonomously classify specific behaviors (Nilsson et al., 2020; Goodwin et al., 2024). It then uses these data as input to create random forest classifiers to label animal behaviors. This approach has been adopted to assess various behaviors such as social interactions (sniffing) (Popik et al., 2024), fear responses (freezing) (Hon et al., 2022), anxiety-related behaviors (head dipping in the elevated plus maze) (Sturman et al., 2020; Bühler et al., 2023), and maternal behaviors (pup retrieval) (Lapp et al., 2023).

The DeepLabCut + SimBA workflow has not yet been used to assess diverse defensive behaviors in response to threat-associated cues. Here, we therefore aimed to evaluate DeepLabCut with SimBA for ethological analysis for this type of data. Specifically, we first used manual scoring to validate that rat displayed defensive behaviors in addition to the commonly observed readouts such as conditioned suppression and freezing elicited by threat-associated cues. Next, we implemented a machine learning workflow to simultaneously quantify defensive behaviors during fear expression. To evaluate the efficacy of utilizing DeepLabCut combined with SimBA, we then compare the obtained scores to the scores from manual annotation.

## 2 Materials and methods

### 2.1 Animals

Adult male (250–300 g) and female (150–200 g) Wistar rats (Charles River, Germany) were pair-housed with a same-sex conspecific in a humidity- and temperature-controlled environment under a reverse light cycle (light on at 7am). All experiments took place during the dark phase. Rats had access to food and water ad libitum and were acclimatized to the facility and handled by experimenters prior to the experiment.

### 2.2 Ethics approval statement

Procedures were approved by the Local Animal Ethics Committee at Linköping university and were in accordance with the EU Directive 2010/63/EU on the protection of animals used for scientific purposes as implemented in Swedish national regulations.

### 2.3 Apparatus

All experiments took place in 16 operant chambers (29.5 cm × 16–32 cm × 21 cm, Med Associates Inc., St. Albans, Vermont) equipped with two levers – active and inactive lever, two receptacles, rod flooring connected to a shock generator, and a speaker inside an individual sound-attenuated cubicle. Chambers were cleaned with chlorine-based disinfectant solution between each session. All chambers were digitally controlled, and numbers of lever presses were automatically recorded by MEDPC-IV software (Med Associates Inc., Fairfax, VT, USA).

### 2.4 Video recording

Behaviors in each chamber were recorded using AXIS M1065-L network cameras with infrared illumination (Axis Communications AB, Lund, Sweden). Videos were recorded at 25 frames per second at 1920 x 1080 resolution. Due to the size of the operant chamber, the camera was placed on the ceiling of each sound-attenuated cubicle to obtain a 45-degree top-down recording that provided sufficient viewing of the whole chamber. Cameras were controlled by MediaRecorder (Version 5, Noldus, Amsterdam, The Netherlands).

### 2.5 Behavioral procedures

**FIGURE 1:**
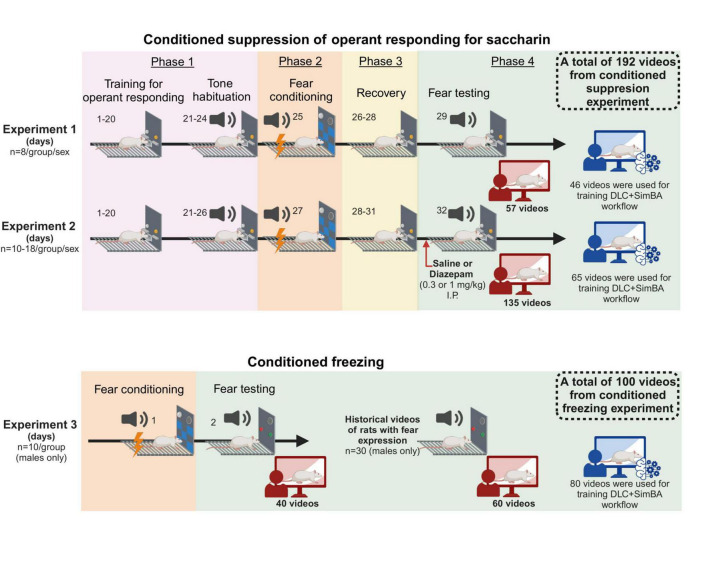
Schematic showing timeline of experiment 1, 2, and 3. In total, 192 videos from conditioned suppression experiment and 100 videos from conditioned freezing experiment were used for training and validating DeepLabCut + SimBA workflow. All schematics were made using Biorender^®^.

A total of 180 male and 128 female rats were used, and three experiments were carried out (Figure 1). In experiment 1 and 2, the setup was optimized for assessment of conditioned suppression of operant responding; in these experiments, the full size of the operant chambers was available to the rats (32 x 29 cm), and operant levers as well as a liquid receptacle were available. Prior to assessment of conditioned suppression of responding, rats were trained on operant responding for saccharin as described below and in the Supplementary material. In experiment 3, the setup was optimized for assessment of freezing; for this, chambers were equipped with dividers that reduced the chamber size to 16–24 × 29 cm, which in preliminary experiments was found to promote freezing behavior. In these experiments, rats had no access to levers nor receptacles and did not undergo any training in the operant chambers prior to fear conditioning.

#### 2.5.1 Experiment 1 and 2

Experiments to assess conditioned suppression consisted of 4 main phases: training, fear conditioning, recovery period, and fear testing (see Supplementary material and Supplementary Figure 1 for details on conditioned suppression of operant responding for saccharin). In experiment 1, we used both male and female rats, and assessed whether defensive behaviors during conditioned suppression were affected by shock intensity. Following training on operant responding and habituation to the tone, rats were equally divided into 4 groups. Each group underwent cued fear conditioning using either 0.4mA, 0.6mA or 0.8mA footshock, or no shock controls (n = 8/group/sex) in chambers with a novel context without lever introduction (i.e., different disinfectant, illumination, and wall pattern from the operant responding context). During conditioning, rats received 3 repeated conditioning trials of 30s tone (conditioned stimulus, CS, 2.9kHz, 70dB) co-terminating with 2s footshock (unconditioned stimulus, US) with a 3 min intertrial interval. After fear acquisition, rats underwent 3 sessions of saccharin self-administration to allow recovery of baseline operant responding rates. On the next day, expression of conditioned fear was tested. For this, the CS was presented with a duration of 2 min, starting at the 3*^rd^* minute of the self-administration session, and in the absence of the US. Videos from fear testing were manually scored and used for training and validating DeepLabCut + SimBA workflow.

In experiment 2, we investigated the effect of diazepam (0.3 and 1 mg/kg) on threat responses, using both male and female rats, under the same protocol as experiment 1 unless stated otherwise. Following training for operant responding and tone habituation, rats underwent fear conditioning using either 0.4 mA or 0.8mA. During the recovery period, rats were habituated to intraperitoneal injections at least twice before fear testing. Once baseline responding rates were stable, rats from each conditioning group were further divided into three subgroups: vehicle control (saline), diazepam 0.3 mg/kg and diazepam 1 mg/kg (*n* = 10–18/subgroup). Rats received an intraperitoneal injection of the respective treatment 15 minutes before testing. Conditioned suppression was then assessed during fear testing and videos were used for training and validating DeepLabCut + SimBA workflow.

#### 2.5.2 Experiment 3

In experiment 3, we investigated the validity of DeepLabCut + SimBA workflow to assess defensive behaviors during cued fear memory testing in a condition without operant responding. This experiment used the same protocol as previously described (Barchiesi et al., 2022). On day 1, male rats were randomly divided into two groups (*n* = 10/group). One group underwent cued fear conditioning using 6 repeated conditioning trials of 30s tone co-terminating with 2s footshocks (30 s CS, 2 s US 0.8 mA, 3 min intertrial interval) while another group was assigned to be a control group, in which rats underwent the same procedures without shock delivery. On day 2, rats were tested for expression of conditioned fear in a novel context (i.e., different disinfectant, illumination, and wall pattern from the conditioning context). After a 5 min initial habituation period, rats were exposed to two CS presentations (30s CS, 2.9kHz, 3 min intertrial interval) without footshock delivery. Videos from fear testing were manually scored, and each video was shortened using FFmpeg module in DeepLabCut into two separate videos: a 30s video recording during the first CS, and 30 s video recording during the second CS. Shortened videos during the second CS were used for training DeepLabCut + SimBA workflow alongside historical 60 annotated videos (30–90 s duration) of male rats that underwent fear memory testing in the same context. Shortened videos during the first CS were used as holdouts for validating the workflow.

### 2.6 Ethological analysis by manual scoring

All videos that were used for training the neural network, building behavior classifiers, and validating DeepLabCut + SimBA workflow were annotated for observable behaviors using a manual scoring function in Ethovision XT software (Version 17, Noldus, Amsterdam, The Netherlands). Each behavior was scored as a mutually exclusive start-stop event. The definitions were as follows:

– Operant: engagement in the operant responding task, either pressing the active lever or retrieving the saccharin reinforcer.
– Grooming: scratching, nibbling, or rubbing on individual’s own body (Spruijt et al., 1992).
– Sniffing: standing on four paws and sniffing on the wall or the grid floor, either with or without locomotor activity.
– Rearing: showing vertical exploration by sniffing while standing on two hind paws, either against the wall or without support (Dielenberg and McGregor, 2001).
– Free-air whisking: non-contact sniffing while the posterior portion of the body is immobile (Towal and Hartmann, 2008).
– Head scanning: oscillating movement of the anterior portion of the body without sniffing while the posterior portion of the body is immobile (Blanchard and Blanchard, 1988; Kavaliers and Choleris, 2001). The minimum bout duration was 1s.
– Freezing: immobility except respiratory movement that lasts at least 1s (Blanchard and Blanchard, 1969).

Following scoring in Ethovision XT, time event plots were checked to ensure that behaviors did not overlap. In videos from experiment 1 and 2, behaviors were annotated during the 2 min CS. In videos from experiment 3, behaviors were annotated during both 30s CS. In historical videos, behaviors were annotated during 30s CSs, 30s before and 30s after the first CS. Time spent on each behavior during the CS period was calculated as a percentage of the CS duration for data analysis. Manual scoring log files were modified using our custom-made python script to correct all file paths and trial times and to standardize annotated terms. The modified log files were later used for building classifiers in SimBA.

### 2.7 Hardware for machine learning models training

Two computers were used for model training:

(1) a HP Z2 Tower G9 Workstation Desktop PC with a 12*^th^* Gen Intel(R) Core i5-12500 3.00 GHz processor, 16.0 GB of RAM, Windows 11, and a NVIDIA GeForce RTX 4060 GPU.
(2) a ThinkStation P360 Tower with an Intel(R) Core i7-10700 2.90 GHz processor, 16.0 GB of RAM, Windows 10, and a NVIDIA GeForce RTX 3060 GPU.

### 2.8 Creating pose estimation data in DeepLabCut

Pose estimation for each video was created using DeepLabCut version 2.3.8 (Mathis et al., 2018). A total of 1,915 frames were extracted from 79 training videos from experiments 1 and 2. An eight-point labeling system (i.e., ear left, ear right, nose, center, lateral left, lateral right, tail base, tail end) was used to match the required inputs for subsequent post-processing step in SimBA (Supplementary Figure 2A). The default 95% of labeled frames were used to train ResNet-50 network, while the remaining 5% were used as a test dataset for neural network evaluation. Based on training statistics, the neural network improves its performance as the number of training iterations increases, reaching a plateau of maximum performance as indicated by training loss at approximately 500,000 training iterations (Supplementary Figure 2B). After experimentation with a variety of number of iterations and shuffles, we decided to use 500,000 training iterations with a shuffle of 1 and batch size of 8 for training the neural network. This resulted in a training error of 3.67 and a testing error of 18.9, which was reduced to a training error of 3.46 and a test error of 13.99 with a p-cutoff of 0.6, a standard probability cutoff that restricts the dataset to confident predictions with a reported likelihood greater than 60% (Popik et al., 2024; Supplementary Figure 2C). Pose estimation data was generated for all videos, and the filtered tracking data were exported as CSV files and used for extracting features in SimBA.

### 2.9 Building random forest behavior classifiers in SimBA

Videos and their corresponding filtered tracking data were imported to SimBA version 1.79.5 (Nilsson et al., 2020). While importing the data into SimBA, Gaussian smoothing was applied over 200ms intervals and the interpolation step was skipped. The distances in the videos were calibrated into pixel per millimeter using the width of the operant chamber (16–32 cm) as a reference value, yielding an approximate 2 pixel per mm value across each video (Supplementary Figure 3A). A series of SimBA iterations (iterations 1–4) with an increasing number of human-annotated videos were created to determine the amount of training data required to construct a sufficiently robust learning model. Outlier correction was applied in iteration 1 with a location correction criterion of 1.5, a movement correction criterion of 1.0, and median as the aggregation method. This step became unnecessary when the accuracy of pose estimation from our DeepLabCut neural network was satisfactory, as indicated by relatively low training and test errors. Therefore, the outlier correction step was skipped in the following iterations.

Our training dataset included 191 videos with 30–120 s duration, which equals a total of 438,000 training frames. The remaining videos that were not included in the training dataset were saved as holdouts for validating the model. The training dataset of iterations 1 and 2 contained only videos from conditioned suppression of operant responding (i.e. experiments 1 and 2). To increase representations of infrequently observed behaviors (i.e., free-air whisking, head scanning, freezing), videos from cued fear testing were added to supplement the training dataset in the subsequent iteration. Therefore, the training dataset of iterations 3 and 4 contained videos from experiments 1–3. All classifiers from iterations 1–4 were evaluated with holdout videos from the conditioned suppression experiments. To examine if our behavioral classifiers were transferable between experimental conditions, we used classifiers from iteration 4, the iteration with the highest performance while assessing videos from experiment 1 and 2, to assess holdout videos from experiment 3. Concurrently, we created iteration 5 that was trained on only videos from experiment 3 and validated the classifiers with holdout videos from the same experiment. The number of videos/frames used as training and validation dataset in each iteration are shown in Table 1.

**TABLE 1:** Number of videos in training and holdout validation dataset of each SimBA iteration.

SimBA iteration	Training dataset					Validation dataset			
	Conditioned suppression experiment (Experiment 1 and 2)		Conditioned freezing experiment (Experiment 3)		Total number of frames	Conditioned suppression experiment (Experiment 1 and 2)		Conditioned freezing experiment (Experiment 3)	
	Number of videos	Number of frames	Number of videos	Number of frames		Number of videos	Number of frames	Number of videos	Number of frames
1	46	162,000	0	0	162,000	147	438,000	0	0
2	81	243,000	0	0	243,000	111	333,000	0	0
3	82	246,000	20	15,000	261,000	110	330,000	0	0
4	111	333,000	80	105,000	438,000	81	243,000	20	15,000
5	0	0	80	105,000	105,000	0	0	20	15,000

SimBA used filtered tracking data to extract 221 features that were divided into 8 categories according to measurement metrics (Supplementary Table 1). To examine how the model used extracted features to classify behavior, we calculated feature permutation importance using a built-in module in SimBA (see more details in Supplementary material and Supplementary Figure 5). Although Shapley additive explanation (SHAP) values were used as an explainability method that indicates contribution of specific features to the model prediction in the seminal SimBA work (Goodwin et al., 2024), we failed to compute SHAP values in our model.

Based on the extracted feature values in each frame, together with behavior labels from Ethovision manual scoring log files, we built behavior classifiers using a random forest model, an ensemble learning model that relies on the majority votes of decision trees for behavioral classification. The model was computed with the following hyperparameters: 600–2,000 random forest estimators, 1–2 minimum sample leaf node, RF_criterion = gini, RF_max_features = sqrt, and test size = 20%. We acknowledged an imbalance of behavior representation in our training data; however, applying oversampling/undersampling in the parameter worsened the performance of machine learning prediction during pilot experimentation. Therefore, no sampling adjustment was set. The minimum bout time was set to 1s for head scanning and freezing classifiers, and 0.2s for all the remaining classifiers.

Mutual exclusivity correction was performed to avoid classified behaviors overlapping in the same frames, with the more frequently detected behaviors being chosen to win any “tiebreakers” (i.e., in case two or more behaviors were equally likely to be classified as present at the same time, the more ubiquitous behavior was favored). Kleinberg smoothing was also leveraged for infrequent behaviors (i.e. grooming, free-air whisking, head scanning, and freezing) in iterations 2 and 3. We discarded this smoothing step in iteration 4 since the representations of these behaviors became sufficient in the training dataset.

### 2.10 Assessing accuracy performance and validating SimBA classifiers

With the analysis of 20% test frames from the training dataset, the accuracy performance for each classifier was measured in SimBA, reported as F1 score. This score was calculated from a harmonic mean of precision, a ratio of true positive frames over all frames classified as positive, and recall, a ratio of true positive frames over all frames that should have been classified as positive. Using a precision-recall curve of each classifier, we determined a discrimination threshold, a threshold of probability at which a given behavior is classified as present (Supplementary Figures 3B–H). The discrimination thresholds were initially selected to maximize accuracy based on F1 score (d_*F*1*max*_). Since our training dataset was relatively small compared to other studies (Lapp et al., 2023; Goodwin et al., 2024; Popik et al., 2024), the thresholds were then adjusted (d_*adj*_) by experimenters examining 2 samples of validating videos that contained each behavior alongside the corresponding probability-time graphs to provide better classification of validation dataset. For classifiers of underrepresented behaviors, we noticed a greater variation among d_*adj*_ during the first manual inspection. Therefore, additional 2–3 sample videos were later examined to calculate mean adjusted discrimination threshold for grooming, free-air whisking, head scanning, and freezing classifiers. Adjustment of discrimination threshold was skipped while validating classifiers from iteration 1 due to an overfitting issue.

To validate SimBA models, classifiers were assigned with both d_*F*1*max*_ and d_*adj*_ while scoring validation videos. Similar to data from manual scoring, data from DeepLabCut + SimBA workflow were analyzed as percentage time spent on each behavior relative to the CS duration. Data from machine learning classification by classifiers assigned with d_*F*1*max*_ and d_*adj*_ were used to determine inter-method reliability by calculating Pearson’s r with data from manual scoring. The flowchart of key steps and a step-by-step guide for implementing DeepLabCut + SimBA workflow to perform ethological analysis of fear expression can be found in Supplementary material and Supplementary Figure 6.

### 2.11 Statistical analysis

All data were analyzed using GraphPad Prism version 10 (GraphPad Software, San Diego, CA) and STATISTICA (StatSoft 13.0 RRID:SCR_014213). Homogeneity of variance was assessed using the Levene test. When no violation of assumption was observed, parametric ANOVA was used, with factors for each analysis indicated in the result section. Data with non-homogeneous variance were analyzed using Mann-Whitney U test or Kruskal-Wallis ANOVA test. Post hoc comparisons were performed using Newman-Keuls test following parametric test and Dunn’s test following nonparametric test. Data from ethological analysis were analyzed using factor analysis to extract factors with a varimax normalized rotation to reduce dimensionality in an unbiased manner. The Pearson correlation coefficient was calculated for inter-method reliability to compare data from DeepLabCut + SimBA workflow with the data from manual scoring. Outliers were excluded using Grubbs’s test. The accepted level of significance for all test was *p* < 0.05.

## 3 Results

### 3.1 Threat-associated cue heightens risk assessment behaviors during fear expression

**FIGURE 2:**
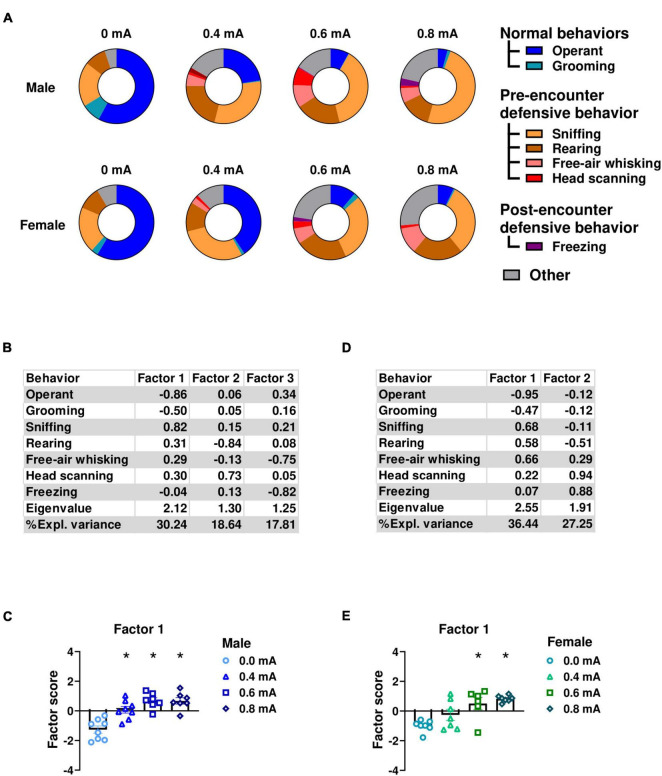
Ethological analysis of observed behaviors during conditioned suppression of operant responding. **(A)** Ethograms showing proportion of time spent on each behavior upon the first CS presentation in fear testing with an effect of footshock intensity during conditioning in males and females. In male rats, factor analysis shows that Factor1 was characterized by decreased operant responding and grooming, and increased sniffing, rearing, and head scanning. **(B)** Factor loading of each behavior in male rats. **(C)** Factor scores for Factor 1 in each conditioning footshock group of male rats. In female rats, factor analysis shows that Factor 1 was characterized by decreased time spent in operant responding and grooming, and increased sniffing, rearing, and free-air whisking. **(D)** Factor loading of each behavior in female rats. **(E)** Factor scores for factor1 in each conditioning footshock group of female rats. All data in this figure were derived from manual annotations. Circles show data from 0.0 mA group. Triangles show data from 0.4 mA group. Squares show data from 0.6 mA group. Diamonds show data from 0.8 mA group. *p < 0.05 compared between different footshock groups. CS, conditioned stimulus. Data are present as mean ± SEM.

In experiment 1, to comprehensively evaluate the range of behaviors elicited by shock-associated cues, we conducted an ethological analysis of the responses to the conditioned stimulus (CS) during fear memory testing in rats subjected to 4 different shock intensities: 0.0mA (no-shock control), 0.4 mA, 0.6 mA, or 0.8 mA electric footshock. This analysis involved manually scoring time spent in operant responding, grooming, sniffing, rearing, free-air whisking, head scanning, and freezing (Figure 2A). We then conducted a factor analysis to identify the underlying behavioral dimension and determine whether heightened defensive behaviors were influenced by threat intensity. Subsequently, we compared factor scores across conditioning groups (0.0, 0.4, 0.6, or 0.8mA). Factor loadings of individual behavioral variables on the extracted factors are shown in Figure 2B for males and in Figure 2D for females.

In male rats, factor1 explained 30.24% of the variance. This factor was characterized by reduced engagement in appetitive and self-care behaviors, evidenced by negative loadings for time spent on operant responding and grooming. Conversely, this factor reflected an increase in defensive behaviors as indicated by positive loadings for time spent in sniffing, rearing, free-air whisking, and head scanning. Subsequently, we investigated whether factor scores of individual rats were affected by shock intensity using one-way ANOVA. Our analysis indicated a significant main effect of Intensity (*F*_(3,26)_ = 17.10; *p* < 0.001; df = 3; Figure 2C), indicating that defensive behaviors are elicited by shock-associated cues as a function of shock intensity. Further post hoc analysis (Newman-Keuls test) showed a significant increase in Factor1 scores in the 0.4, 0.6, and 0.8 mA compared to 0.0 mA groups (*p* < 0.001).

Similar findings were found in female rats. Factor analysis identified Factor1 that accounted for the highest proportion of variance (36.44%) in the data. This factor was characterized by reduced time allocation for operant responding and grooming as indicated by negative loadings. In contrast, engagement in defensive behaviors such as sniffing, rearing, free-air whisking, and head scanning demonstrated positive loadings on the same factor (Figure 2D). Similarly to the analysis in the male data, one-way ANOVA showed a significant effect of intensity on factor scores for Factor 1 (*F*_(3,23)_ = 8.69; *p* < 0.001; df = 3; Figure 2E), indicating that female defensive behaviors are also elicited by shock-associated cues as a function of shock intensity. Post hoc analysis (Newman-Keuls test) showed that the 0.6 and the 0.8 mA significantly differed from the 0.0 mA group (*p* < 0.01). Collectively, these data indicate that both male and female rats show a reduction in consummatory behavior alongside an increase in defensive behaviors in response to the CS.

### 3.2 Heightened risk assessment behaviors are reduced by diazepam

**FIGURE 3:**
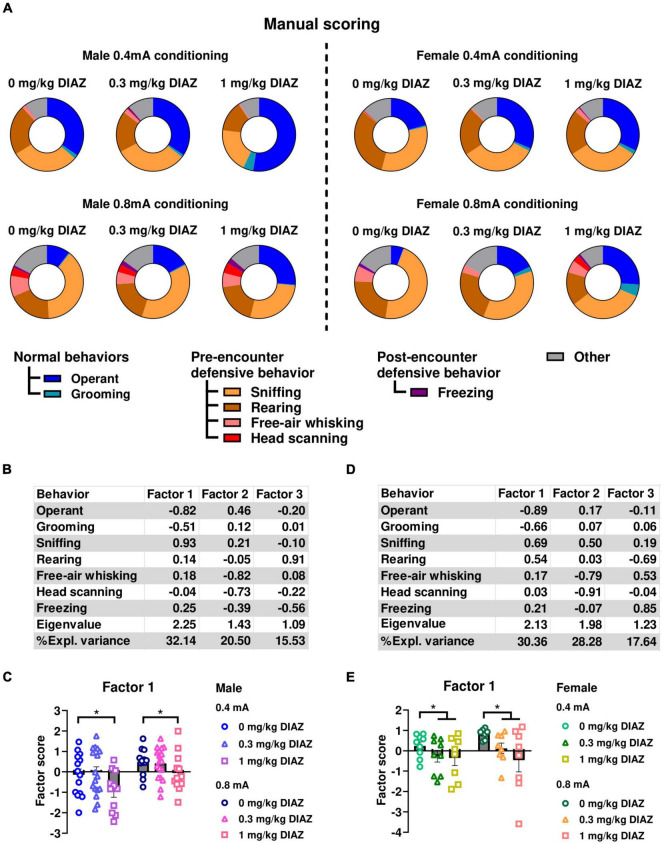
Effects of diazepam on defensive behaviors when fear is assessed by conditioned suppression. **(A)** Ethograms showing proportion of time spent on each behavior upon the first CS presentation in fear testing with an effect of diazepam in males (left panels) and females (right panels). **(B,C)** Factor analysis shows that Factor1 in males was characterized by negative loadings from time spent on operant responding, grooming and positive loading from time spent on sniffing. **(B)** Factor loading of each behavior of male rats. **(C)** Factor scores for Factor1 in each group of male rats. **(D,E)** Factor analysis shows that Factor1 in females was characterized by negative loadings from time spent on operant responding and grooming, and positive loadings from time spent on sniffing and rearing. **(D)** Factor loading of each behavior of female rats. **(E)** Factor scores for Factor1 in each group of female rats. Circles show data from 0.0 mg/kg diazepam treatment group, triangles show data from 0.3 mg/kg diazepam treatment group, squares show data from 1 mg/kg diazepam treatment group. All data in this figure were derived from manual annotations. CS, conditioned stimulus; DIAZ, diazepam. *p < 0.05 compared between diazepam treatment groups. Data are present as mean ± SEM.

To further validate that the ethological readouts evaluated in experiment 1 accurately depict defensive behaviors, we conducted experiment 2 to examine the effect of the anxiolytic diazepam on these behaviors. In this experiment, male and female rats underwent a conditioning session with either 0.4, or 0.8 mA electric footshock, and were tested one week later for fear expression after receiving either 0.3 mg/kg, 1 mg/kg diazepam, or vehicle. Ethograms of observed behaviors in male rats during the first CS presentation are shown in Figure 3A. Similar to experiment 1, in male rats, factor analysis identified Factor1, characterized by negative loadings from time spent on operant responding and grooming, alongside positive loadings from sniffing. This factor accounted for a significant proportion of the variance (32.14%) (Figure 3B). Rearing, free-air whisking, and head scanning loaded on two additional factors that accounted for smaller variance components, 20.50% and 15.53%, respectively. We conducted a two-way ANOVA, using Factor1 scores as the dependent variable, with diazepam dose and shock intensity as categorical factors. The analysis showed a significant main effect of dose (*F*_(2,80)_ = 4.62; *p* = 0.013; df = 2), showing that diazepam had a significant effect to reduce defensive behaviors. We also found a significant main effect of Intensity (*F*_(1,80)_ = 10.12; *p* = 0.002; df = 1), but not a significant interaction of Intensity X Dose (*F*_(2,80)_ = 0.41; *p* = 0.7; df = 1; Figure 3C). Post hoc comparison using the Newman-Keuls test showed that the main effect of dose was largely driven by the 1mg/kg dose of diazepam, as rats exposed to 1 mg/kg diazepam significantly decreased Factor1 scores compared to 0.3 mg/kg diazepam (*p* = 0.03) and vehicle (*p* = 0.01), while no significant effect vs. vehicle was seen in the 0.3 mg/kg dose group.

The effect of diazepam to reduce defensive behaviors was also observed in females (Figure 3A, right panels). Similar to the findings in the male data, the factor analysis on behaviors exhibited during the first CS identified a factor characterized by a decrease in time allocated to operant responding and grooming, associated with an increase in sniffing and rearing (Figure 3D). This factor explained the highest proportion of variance in the female data, 30.36%. Time spent on free-air whisking and head scanning, and freezing loaded on two additional factors, which accounted for a smaller proportion of the variance, 28.28% and 17.64%, respectively. Two-way ANOVA using Factor1 scores as dependent variable with diazepam dose and shock intensity as categorical factors indicated a significant main effect of dose (*F*_(2,43)_ = 3.95; *p* = 0.027; df = 2; Figure 3E), suggesting that diazepam influenced defensive behaviors in female rats. No main effect of Intensity (*F*_(1,43)_ = 1.09; *p* = 0.30; df = 1), or significant interaction Dose X Intensity (*F*_(2,43)_ = 0.58; *p* = 0.58; df = 2) were found. Post hoc analysis using Newman-Keuls test showed that diazepam at the dose of 1 mg/kg showed a trend of decreased Factor1 scores compared to the vehicle group in the 0.8 mA conditioned females (p = 0.09). Collectively, these results demonstrate that diazepam reduced defensive behaviors in response to shock-associated cues in both male and female rats, and suggest that our ethological readouts have a good predictive validity.

### 3.3 Development of DeepLabCut + SimBA workflow for ethological analysis of fear responses

Machine-learning tools have been recently used to automate simultaneously quantification of multiple behaviors in rats but have not yet been applied to the type of fear-related experiments conducted here. We therefore developed a workflow using DeepLabCut and SimBA to assess fear-related behaviors. A series of SimBA iterations were developed, and the accuracy performance of classifiers was evaluated and reported as precision, recall, and F1 score (Table 2). After determining discrimination thresholds, classifiers were validated by scoring validation videos. The obtained scores were then used for calculating Pearson’s correlation with scores from standard manual scoring to assess inter-method reliability.

**TABLE 2:** Validation of DeepLabCut + SimBA workflow using videos from conditioned suppression experiment (experiment 1 and 2).

Classifier	SimBA iteration	Number of frames in training dataset	Random forest estimators	Precision	Recall	F1	d_F1max_	r	*p*-value	d_adj_	r	*p*-value
Operant	1	41634	2000	0.98	0.96	0.97	0.437	0.32	<0.001	N/A	N/A	N/A
	2	58077	1000	0.95	0.85	0.90	0.358	**0.92**	**<0.001**	0.312	**0.92**	**<0.001**
	3	57159	1000	0.96	0.85	0.90	0.363	**0.91**	**<0.001**	0.312	**0.93**	**<0.001**
	4	74898	1000	0.97	0.83	0.89	0.330	**0.94**	**<0.001**	0.270	**0.95**	**<0.001**
Grooming	1	2592	2000	0.98	0.94	0.96	0.437	0.00	N/A	N/A	N/A	N/A
	2	7047	600	0.99	0.70	0.82	0.231	0.54	<0.001	0.216	0.54	<0.001
	3	6264	600	0.99	0.67	0.80	0.208	0.67	<0.001	0.216	0.68	<0.001
	4	8760	700	1.00	0.64	0.78	0.190	0.62	<0.001	0.209	0.66	<0.001
Sniffing	1	47952	2000	0.95	0.93	0.94	0.439	0.18	0.028	N/A	N/A	N/A
	2	70956	1000	0.99	0.83	0.90	0.409	**0.72**	**<0.001**	0.387	**0.71**	**<0.001**
	3	73341	1000	0.99	0.78	0.88	0.387	**0.76**	**<0.001**	0.387	**0.76**	**<0.001**
	4	154176	1000	0.99	0.91	0.95	0.434	**0.91**	**<0.001**	0.412	**0.91**	**<0.001**
Rearing	1	26892	2000	0.94	0.89	0.92	0.375	0.21	0.010	N/A	N/A	N/A
	2	38880	750	0.98	0.63	0.77	0.341	**0.81**	**<0.001**	0.316	**0.82**	**<0.001**
	3	43587	750	0.99	0.67	0.76	0.333	**0.86**	**<0.001**	0.316	**0.86**	**<0.001**
	4	77964	750	0.99	0.73	0.84	0.339	**0.88**	**<0.001**	0.295	**0.89**	**<0.001**
Free-air whisking	1	7938	2000	0.95	0.84	0.89	0.343	0.01	0.856	N/A	N/A	N/A
	2	14580	600	0.99	0.51	0.67	0.227	0.57	<0.001	0.180	0.63	<0.001
	3	18531	600	0.99	0.50	0.67	0.230	0.54	<0.001	0.180	0.67	<0.001
	4	28470	750	1.00	0.56	0.72	0.217	**0.83**	**<0.001**	0.180	**0.85**	**<0.001**
Head scanning	1	2592	2000	0.99	0.97	0.98	0.366	0.00	N/A	N/A	N/A	N/A
	2	6318	750	1.00	0.74	0.85	0.219	0.20	0.038	0.150	0.09	0.328
	3	6786	750	1.00	0.71	0.83	0.185	−0.01	0.879	0.150	0.16	0.091
	4	8760	750	1.00	0.68	0.81	0.175	**0.76**	**<0.001**	0.150	**0.71**	**<0.001**
Freezing	1	1296	2000	0.95	0.89	0.92	0.364	0.00	N/A	N/A	N/A	N/A
	2	3402	600	0.99	0.60	0.75	0.173	0.53	<0.001	0.142	0.38	<0.001
	3	8091	600	0.99	0.73	0.84	0.205	0.00	0.000	0.142	0.12	0.220
	4	19272	700	1.00	0.83	0.91	0.269	−0.02	0.848	0.142	−0.02	0.854

Accuracy parameters (i.e., Precision, Recall, F1) within the 20% holdout frames were calculated from SimBA. Discrimination thresholds at maximum F1 value (d_F1max_) and after adjustment (d_adj_) were applied to score behaviors on validation videos and Pearson correlations between scores from DeepLabCut + SimBA workflow and manual scoring were calculated. Using d_adj_ slightly improved accuracy in general but it was insufficient to improve accuracy for rarely observed behaviors. Since overfitting was observed from behavior classification using Iteration 1, d_adj_ was not determined for classifiers in this iteration. Numbers with underline: an outlier was excluded while calculating Pearson’s correlation. Number in bold: a significant strong correlation (Pearson’s *r* > 0.7).

**FIGURE 4:**
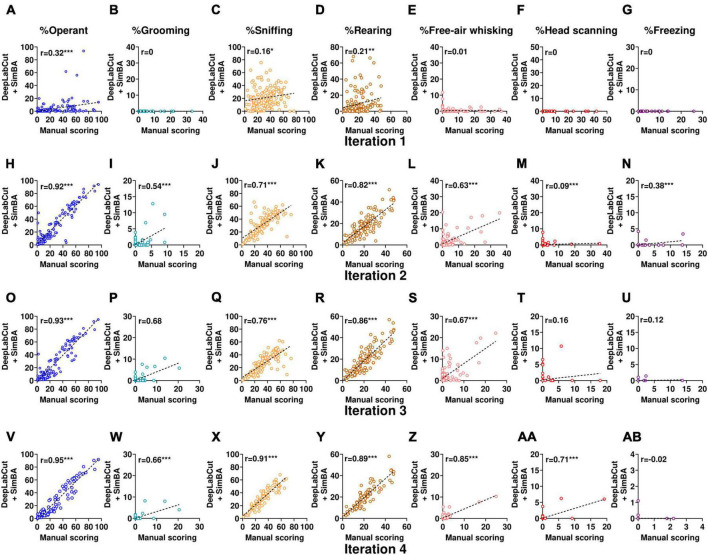
Comparison of scores from DeepLabCut + SimBA workflow with scores from manual scoring while evaluating holdout videos from conditioned suppression experiment (experiment 1 and 2). Correlation analyses of holdout videos that were not included in the training dataset using classifiers from iteration 1 **(A–I)**, iteration 2 **(H–N)**, iteration 3 **(O–U)**, and iteration 4 **(V–AB)**. Outliers were excluded from correlation analyses of head scanning classifier in iteration 3 and free-air whisking and freezing classifiers in iteration 4. The axes represent the percentage of time spent on each behavior relative to the CS duration. Note that head scanning and freezing infrequently occurred in the validation dataset. CS, conditioned stimulus. *p < 0.05; **p < 0.01; ***p < 0.001 compared between scoring methods.

In the first iteration, the overall performance of SimBA classifiers was high as indicated by F1 score > 0.89 for all classifiers. However, while evaluating the validation videos, we observed near zero correlations between the obtained scores from our SimBA model and the scores derived from manual scoring (Figures 4A–G). This suggests that the model only accurately classified training data but was unable to classify other data, demonstrating overfitting of the model. To overcome this problem, subsequent SimBA iterations were then developed by lowering the number of estimators and adding more training datasets (Table 2). The following models attained moderate to high accuracy performance (F1 score ranging from 0.671 to 0.902) and showed better generalizability to score validation videos (Figures 4H–AB).

When the behaviors were commonly represented (> 30,000 frames in the training dataset), such as operant responding, sniffing, and rearing, scores from SimBA classifiers strongly correlated with manual annotation (*r* = 0.72–0.95, all with *p* < 0.001; Table 2), demonstrating high reliability between scoring methods. When the behaviors were moderately frequent (∼15,000–25,000 frames in the training dataset), such as grooming and free-air whisking, scores from the classifier showed a moderate to strong correlations with manual annotation as indicated by Pearson’s r (grooming classifier: r = 0.54, 0.67, 0.62; free-air whisking classifier: r = 0.57, 0.53, 0.83 in iteration 2, 3, 4, respectively; all with p < 0.001. Interestingly, in cases where the training dataset was relatively limited (i.e., free-air whisking in iteration 2), employing d_*adj*_ rather than d_*F*1*max*_ for the classifier resulted in performance improvement, as indicated by an increased Pearson’s r (from *r* = 0.57 to 0.63; *p* < 0.001; Figure 4L and Table 2). However, when the number of training dataset was high (i.e., iteration 4), assigning d_*adj*_ only marginally improved the accuracy for detecting free-air whisking (from *r* = 0.83 to 0.85, both with *p* < 0.001; Figure 4Z). This highlights that increasing the training dataset is the major parameter for improving classifier performance.

When the behaviors were underrepresented in the training dataset (< 15,000 frames), classifiers showed weak to moderate inter-method reliability: for grooming (*r* = 0.54–0.66; all with *p* < 0.001; Figures 4I, P, W), head scanning (*r* = 0.09, 0.16, 0.71; *p* = 0.33, 0.09, < 0.001; Figures 4M, T, AA) and freezing (*r* = 0.38, 0.12, −0.02; *p* < 0.001, 0.22, 0.85; Figures 4N, U, AB). Notably, the performance evaluation of head scanning and freezing classifiers remained non-optimal due to the infrequent occurrence of these behaviors in the holdout videos used for classifier validation. Besides, low inter-method reliability among these classifiers may be because outlier data were excluded while calculating Pearson’s *r*.

### 3.4 Transferability of SimBA model to detect defensive behaviors

To evaluate the transferability of our SimBA model to a different experimental condition, we used the classifiers from iteration 4, which had the largest training dataset, to assess validation videos from the conditioned freezing experiments. Comparing the scores derived from the SimBA model with manual annotation indicated a significantly high inter-method reliability for rearing and freezing (*r* = 0.93 and 0.89, respectively, both with *p* < 0.001; Table 3 and Supplementary Figures 4B, E). Although the sniffing classifier was trained on the highest number of frames (154,176) among all classifiers in this iteration, its performance in SimBA only showed a moderate correlation with manual annotation (*r* = 0.46, *p* < 0.05; Supplementary Figure 4A). Furthermore, the model detected free-air whisking less frequently than manual annotation, resulting in a non-significant correlation between the two scoring methods (*r* = 0.20; *p* = 0.39; Supplementary Figure 4C).

**TABLE 3:** Validation of DeepLabCut + SimBA workflow using videos from conditioned freezing experiment (experiment 3).

Classifier	SimBA iteration	Number of frames in training dataset	Random forest estimators	Precision	Recall	F1	d_F1max_	r	*p*-value	d_adj_	r	*p*-value
Grooming	4	8760	700	1.00	0.64	0.78	0.190	N/A	N/A	N/A	N/A	N/A
	5	1050	600	1.00	0.66	0.80	0.178	N/A	N/A	N/A	N/A	N/A
Sniffing	4	154176	1000	0.99	0.91	0.95	0.434	0.42	0.068	0.412	0.46	0.040
	5	54075	1000	0.97	0.98	0.98	0.541	**0.80**	**<0.001**	0.590	**0.81**	**<0.001**
Rearing	4	77964	750	0.99	0.73	0.84	0.339	**0.90**	**<0.001**	0.295	**0.93**	**<0.001**
	5	21525	750	0.99	0.87	0.93	0.401	**0.95**	**<0.001**	0.401	**0.93**	**<0.001**
Free-air whisking	4	28470	750	1.00	0.56	0.72	0.217	0.27	0.255	0.180	0.20	0.394
	5	8190	600	1.00	0.72	0.83	0.230	0.23	0.323	0.145	0.37	0.106
Head scanning	4	8760	750	1.00	0.68	0.81	0.175	0.00	N/A	0.150	0.00	N/A
	5	1155	600	1.00	0.85	0.92	0.253	0.00	N/A	0.021	−0.24	0.317
Freezing	4	19272	700	1.00	0.83	0.91	0.269	**0.92**	**<0.001**	0.142	**0.90**	**<0.001**
	5	14595	600	0.99	0.95	0.97	0.340	**0.89**	**<0.001**	0.260	**0.95**	**<0.001**

Accuracy parameters (Precision, Recall, F1) within the 20% test frames were calculated from SimBA. Discrimination threshold at maximum F1 (d_F1max_) and adjusted discrimination thresholds (d_adj_) was applied on classifiers for behavior classification of non-training validating videos before calculating Pearson’s correlation coefficient between DeepLabCut + SimBA scoring and manual scoring. Scoring obtained from iteration 5 that was only trained on videos from experiment 3 has higher accuracy (i.e. Higher F1 and higher Pearson’s correlation coefficient) than those from iteration 4 that was trained on videos from all experiment. Number in bold: a significant strong correlation (Pearson’s *r* > 0.7).

Notably, grooming was absent in the validation videos, which prevented the assessment of this classifier’s performance. Overall, these findings suggest that SimBA classifiers demonstrate an acceptable accuracy for detecting frequently occurring behaviors but are less reliable for detecting infrequent behaviors in videos recorded from different experimental condition.

We then examined whether behavioral classification achieves higher accuracy when training data is exclusively gathered under identical conditions. To assess this, we compared the performance of iteration 4, trained on dataset from conditioned suppression experiments and conditioned freezing experiments (experiment 1 and 2) with that of iteration 5, exclusively trained on videos from conditioned freezing experiments (experiment 3). Classifiers in iteration 5 reported satisfactory accuracy, ranging from F1 score = 0.83 (free-air whisking) to 0.977 (sniffing). When comparing between iteration 4 and 5, we found that iteration 5 showed better performance than iteration 4 as indicated by higher Pearson’s correlation coefficients (Table 3 and Supplementary Figures 4F–J). Specifically, scores from SimBA iteration 5 and manual scoring showed significant correlations with manual annotations for sniffing, rearing, and freezing, with Pearson’s *r* = 0.81, 0.93, and 0.95, respectively (all with *p* < 0.001). The free-air whisking classifier in iteration 5 detected this behavior more frequently than that in iteration 4, yet the detection remained less frequent than manual annotation (*r* = 0.37; *p* = 0.11). Compared to manual annotations, head scanning was underestimated in both iterations (Supplementary Figures 4D, I). This may be partly due to an apparent low number of training frames (1155 frames; Table 3). Collectively, our findings suggest that behavior classifiers have better scoring performances when the model is trained from videos with the same experimental condition than when the model is trained from videos with different condition.

### 3.5 DeepLabCut + SimBA workflow yield similar behavior findings as manual scoring

**FIGURE 5:**
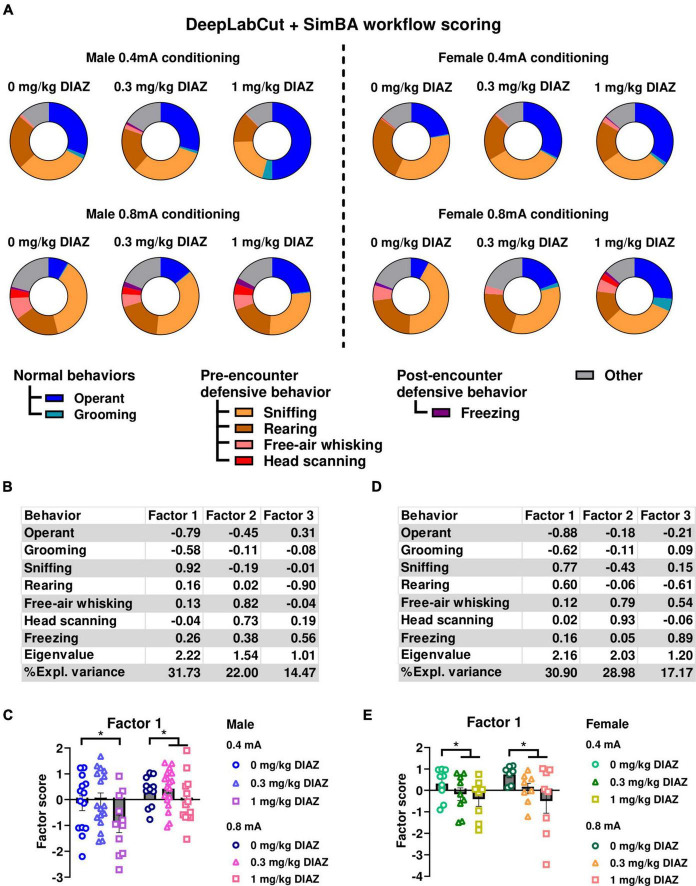
Ethological analysis of behaviors in conditioned suppression experiment (experiment 2) using data derived from DeepLabCut + SimBA workflow. **(A)** Ethograms showing proportion of time spent on each behavior upon the first CS presentation in fear testing with an effect of diazepam in males (left panels) and females (right panels). All data in this figure were annotated using SimBA model that was trained on the highest number of training dataset (iteration 4). Factor analysis shows that Factor1 in males was largely characterized by negative loadings from time spent on operant responding, grooming and positive loading from sniffing. **(B)** Factor loading of each behavior of male rats. **(C)** Factor scores for Factor 1 in each group of male rats. Factor analysis shows that Factor1 in females was characterized by negative loadings from time spent in operant responding and grooming, and positive loadings from time spent sniffing and rearing. **(D)** Factor loading of each behavior of female rats. **(E)** Factor scores for factor1 in each group of female rats. Circles show data from 0.0 mg/kg diazepam treatment group, triangles show data from 0.3 mg/kg diazepam treatment group, squares show data from 1 mg/kg diazepam treatment group. Note that these analyses were performed using both training and validation dataset of the SimBA model. CS: conditioned stimulus. DIAZ: diazepam. *p < 0.05 compared between treatment groups. Data are present as mean ± SEM.

To underscore the efficacy of DeepLabCut + SimBA workflow in ethological analysis, we made ethograms and conducted a factor analysis using the data acquired through DeepLabCut + SimBA model (iteration 4) from videos with conditioned suppression of operant responding (experiment 2). Our findings indicate that the ethograms generated through the DeepLabCut + SimBA workflow are comparable to those generated through manual scoring (Figure 5A, compared to Figure 3A). Furthermore, factor analysis yielded similar results when compared to the manual scoring. Specifically, in male rats, Factor1 explained the highest proportion of variances (31.73%) in the data. Factor1 was characterized by negative loading from time allocation for operant responding and grooming together with positive loading from time allocation for sniffing (Figure 5B). Similar to what found with manual scoring (Figure 3C) two-way ANOVA shows a significant main effect of Dose [*F*_(2, 80)_ = 4.31; *p* = 0.017; df = 2; Figure 5C], and of Intensity [*F*_(1, 80)_ = 9.20; *p* = 0.003; df = 1] with no significant interaction [Intensity X Dose; *F*_(2, 80)_ = 0.48; *p* = 0.620; df = 2].

Likewise, in female rats, Factor1, a factor that explained the highest variance in the data (30.90%), had negative loadings from time allocation for operant responding and grooming, but positive loadings from time allocation for risk assessment (i.e., sniffing and rearing; Figure 5D). We conducted two-way ANOVA using Factor1 score as a dependent variable and diazepam dose and intensity as categorical factors. Consistent with the analysis using manual scoring data, we observed a significant main effect of Dose [*F*_(2, 43)_ = 4.24; *p* = 0.021; df = 2; Figure 5E compared to Figure 3E]. No main effect of Intensity [*F*_(1,43)_ = 0.54; *p* = 0.467; df = 1], or significant interaction Dose X Intensity [*F*_(2,43)_ = 0.29; *p* = 0.750; df = 2] were found.

**FIGURE 6:**
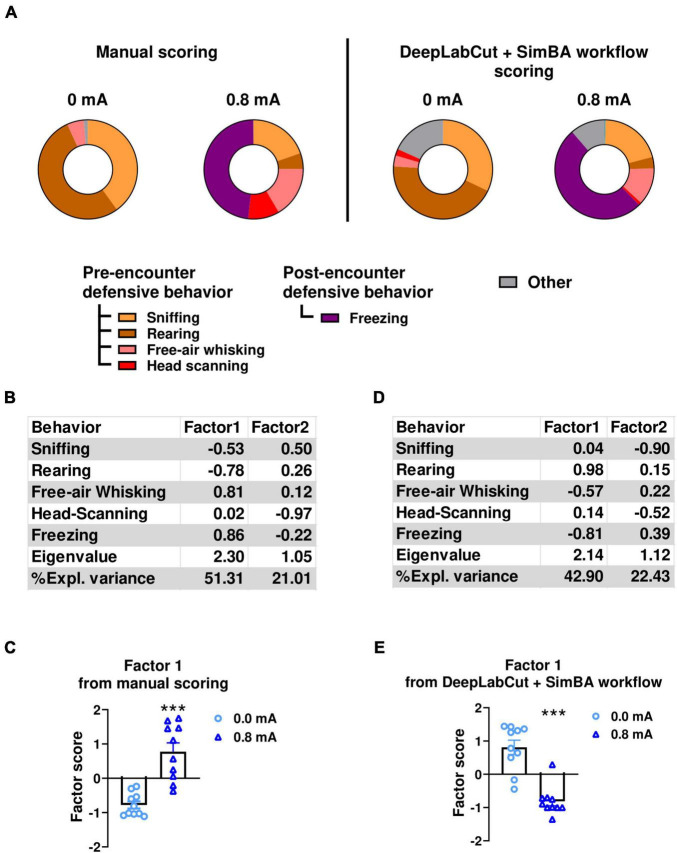
Comparison of ethological analysis of behaviors in conditioned freezing experiment (experiment 3) using data from two scoring methods. **(A)** Ethograms showing proportion of time spent on each behavior upon the first CS presentation in fear testing. These ethograms were derived from manual scoring (left panels) and SimBA model that were trained from only videos from conditioned freezing experiment (iteration 5) (right panels). Factor analyses of observed behaviors using data from manual scoring **(B,C)** and from DeepLabCut + SimBA workflow **(D,E)**. **(B)** Factor loading of each behavior using data from manual scoring. **(C)** Factor scores for Factor1 in 0.8mA-conditioned rats vs. no-shock controls. Factor analysis shows that Factor1 derived from manual scoring data was characterized by negative loading from time spent in sniffing and rearing and positive loading from free-air whisking and freezing. **(D)** Factor loading of each behavior using data from DeepLabCut + SimBA workflow. **(E)** Factor scores for Factor1 in 0.8 mA-conditioned rats vs. no-shock controls. Circles show data from 0.0 mA group (no shock). Triangles show data from 0.8mA-conditioned group. Factor analysis shows that Factor1 derived from DeepLabCut + SimBA workflow had negative loading from time spent free-air whisking and freezing together with positive loading by time spent rearing. Note that these analyses were performed using videos that were not included in training dataset of any SimBA model. CS, conditioned stimulus. ***p < 0.001 compared between groups. Data are present as mean ± SEM.

These findings indicate that the results obtained from DeepLabCut + SimBA workflow align closely with those derived from manual scoring. However, it is important to note that a substantial portion of the videos, nearly half, were incorporated into the training dataset, potentially introducing a positive bias due to overfitting. To address this, we also compared data from manual scoring with that obtained from the DeepLabCut + SimBA workflow (iteration 5) in experiment 3 (Figure 6A). In this experiment, the videos used for scoring were not included in the model training dataset. This approach ensures that the similarities and differences observed in the ethograms are not influenced by the training data.

Factor analyses were then performed from dataset obtained from DeepLabCut + SimBA workflow and manual scoring. Two factors were extracted from the manually scored data (Figure 6B). In particular, Factor1 was characterized by negative loadings from time spent on sniffing and rearing and positive loadings from time spent in free-air whisking and freezing, explaining 51.31% of the variance. One-way ANOVA analysis on Factor1 scores showed a significant increase in Factor1 scores in the 0.8mA group compared to the 0mA group [0 vs. 0.8mA; F_(1,18)_ = 70.5; *p* < 0.001; df = 1; Figure 6C]. A similar pattern of extracted factors and factor loadings was found when a factor analysis was conducted using the scores from DeepLabCut + SimBA workflow. Specifically, time spent on free-air whisking and freezing showed negative loadings, while time spent in rearing showed positive loading positively in factor1 (Figure 6D). This factor accounted for the highest proportion of variance (42.90%). Comparable to manual scoring, one-way ANOVA conducted with data from the DeepLabCut + SimBA workflow showed a significant difference in Factor1 score between group (0 vs. 0.8mA *F*_(1,18)_ = 40.3; *p* < 0.001; df = 1; Figure 6E). Collectively, these findings indicate that the DeepLabCut + SimBA workflow was able to perform ethological analysis of a broad range of defensive behaviors similar to traditional manual scoring.

## 4 Discussion

Expanding on prior research (Blanchard and Blanchard, 1988; Fanselow et al., 1988; Hoffman et al., 2022), our study emphasizes the importance of investigating defensive behaviors within a broader, ethological framework. Our findings indicate that both male and female rats exhibit defensive behaviors when exposed to fear-associated cues. This shows that, besides freezing and conditioned suppression, these responses could serve as additional indicators of fear and fear-related pathologies. Moreover, we demonstrate that DeepLabCut in conjunction with SimBA can effectively measure these behaviors, thereby addressing a significant challenge in evaluating ethological behaviors.

Our results indicate that, in response to a fear-associated cue, rats reduce the time spent on operant responding for a high-value reinforcer and on self-care activities like grooming. This aligns with previous studies showing conditioned suppression of food or water intake in response to a tone associated with a footshock (Estes and Skinner, 1941; Annau and Kamin, 1961; Bouton and Bolles, 1980). We observed that when presented with a fear-associated cue, rats shift their attention to risk-assessment behaviors such as rearing, sniffing, and head scanning. The prevalence of these behaviors increases with the intensity of footshocks, and is reduced by the anxiolytic drug diazepam, providing initial support for predictive validity. Our observations also highlight the rich repertoire of defensive behaviors, underscoring the value of taking a broad range of behaviors into account in studies aimed at furthering the understanding of fear learning and memory mechanisms.

Notably, under the conditions used in experiment 1 and 2, we observed robust fear responses as supported by a suppression of operant responding, and reversal of this effect by diazepam, yet we saw minimal freezing episodes. This result may be explained by the conditions in these experiments, where rats are placed in a large chamber, a setup that likely favors behaviors associated with vigilance or risk assessment (Bolles and Collier, 1976). In line with this hypothesis, we observed robust freezing behaviors in response to the fear-associated cue in experiment 3, when rats were tested for fear expression in smaller chambers without access to operant levers of reinforcer delivery. These findings suggest that the absence of freezing does not necessarily indicate that fear learning has not occurred, highlighting the value of evaluating a comprehensive ethological profile when assessing fear-associated responses.

A limitation of our paradigm was that the camera recording was from semi-top view, which prevented accurate measurement of cued-induced locomotion (Le et al., 2023; Chu et al., 2024) and darting (Gruene et al., 2015). Additionally, other fear responses identified in previous rodent studies, such as jumping (Fadok et al., 2017) and tail rattling (Salay et al., 2018), were not detected in any of our settings. This discrepancy may be due to different protocols and species.

Analyzing complex behaviors has proven challenging due to limited sensitivity of available commercial software and the labor-intensive nature of manual scoring. Moreover, manual scoring is susceptible to low inter-rater reliability (Kafkafi et al., 2018). Recent advances in computer vision and machine learning offer promising tools to achieve human-level accuracy and standardizing behavioral assessments (Datta et al., 2019). For instance, social interaction in rats or mice can now be reliably evaluated using DeepLabCut together with SimBA (Lapp et al., 2023; Goodwin et al., 2024; Popik et al., 2024). Furthermore, a recent study comparing DeepLabCut + SimBA workflow with manual scoring indicated that this combination provides accurate quantification of grooming time but does not reliably measure grooming bouts (Correia et al., 2024), suggesting that the accuracy of this approach depends on the specific behavior being assessed.

In our study, we evaluated whether DeepLabCut combined with SimBA could perform ethological analyses of behavioral responses to fear-associated cues as effectively as manual scoring. To this end, we tested different parameters in a series of SimBA iterations, including the number of training frames and number of estimators. We found that the use of a default setting of 2,000 estimators with a relatively low number of training frames leads to overfitting (iteration 1). By optimizing the estimator to 600–1,000 and increasing the number of training frames containing well-represented behaviors to at least 30,000 frames, we achieved consistently high accuracy (F1 > 0.75) and high inter-method reliability (*r* > 0.7) during the cross-validation. Our optimization aligns with a study from Lapp et al., which reported a range of 1000–1500 estimators for 84,000–750,000 training frames to identify maternal behaviors in rats (Lapp et al., 2023). In contrast, we reported lower amounts of training data compared to the seminal paper describing SimBA (Goodwin et al., 2024). This indicates that the required number of training datasets may be dependent on the experimental conditions and behaviors evaluated. In line with this hypothesis, we found that only 21,525 training frames were sufficient to train the rearing classifier in iteration 5 to achieve satisfactory accuracy when assessed videos from the conditioned freezing experiment, whereas more than 78,000 training frames may be needed to train a classifier for the same behavior in iteration 4 to match the same level of accuracy in the larger chamber used for conditioned suppression of operant responding. Together, this suggests the need for testing the optimal number of estimators and training frames before analysis.

Additionally, we also tested whether similar behaviors can be reliably detected under varying experimental conditions. The ability to transfer the learning model would eliminate the need to retrain DeepLabCut + SimBA when experimental conditions change. SimBA model is hypothesized to have better transferability than other machine learning approaches since features are extracted from a body point-labeling system, providing more flexibility for behavior detection across different setups (von Ziegler et al., 2021). Our findings show that SimBA classifiers in iteration 4 exhibit satisfactory accuracy in detecting rearing in all experimental conditions. However, an accurate detection of other well-represented behaviors such as sniffing was limited to the conditioned suppression experiment, indicating that the chamber size plays a role in the representation of such behavior. To our knowledge, this study represents the first investigation into the transferability of DeepLabCut + SimBA workflow across fear-related experimental conditions. Goodwin et al., developed separate classifiers to identify similar social behaviors across four different resident-intruder datasets with a comparison of classifiers through interpretability tools such as Sharpley additive explanation (SHAP) value (Goodwin et al., 2024). However, their study did not evaluate whether classifiers trained on videos from one experimental condition effectively transferred to videos from another condition. Previous research has evaluated the performance of learning models for automated behavioral recognition across setups using alternative deep learning-based methods such as end-to-end system, demonstrating a lack of model transferability (van Dam et al., 2020). Collectively, these findings suggest that DeepLabCut + SimBA may offer enhanced transferability compared to other deep learning methods (i.e., end-to-end systems). However, it is important to note that all behavior classifiers from DeepLabCut + SimBA may not always transfer to different conditions reliably.

Another challenge we faced in developing the machine learning model for ethological analysis was dealing with imbalance in the representation of different behaviors, similar to previous studies (Nilsson et al., 2020; Popik et al., 2024). Specifically, behaviors like free-air whisking and head scanning were relatively infrequent in our data. While increasing frames containing such behaviors in the model training dataset is a cornerstone to improve classifier performance, we acknowledge that building classifiers may require more experimentation with oversampling-undersampling parameters without worsening accuracy by introducing meaningless new frames or removing important frames (Krawczyk, 2016).

In conclusion, our data support the utility of evaluating a large panel of ethological behaviors as a readout of conditioned fear. Moreover, our study demonstrates the utility of DeepLabCut + SimBA workflow in ethological analysis of complex defensive behaviors, albeit demanding significant numbers of training resources. Together, this approach holds a potential for decoding underlying mechanisms of a different spectrum of fear learning and memory.

## Data availability statement

The datasets presented in this study can be found in online repositories. The names of the repository/repositories and accession number(s) can be found below: https://osf.io/yj7cb/?view_only=f01e4a3969ce46a28f41ca50683208c8.

## Ethics statement

Procedures were approved by the Local Animal Ethics Committee at Linköping University and were in accordance with the EU Directive 2010/63/EU on the protection of animals used for scientific purposes as implemented in Swedish national regulations.

## Author contributions

KC: Data curation, Investigation, Methodology, Software, Visualization, Writing−original draft, Writing−review and editing. YF: Investigation, Methodology, Validation, Writing−review and editing. TW: Data curation, Investigation, Methodology, Software, Validation, Writing−original draft, Writing−review and editing. LF: Investigation, Writing−review and editing. TK: Investigation, Writing−review and editing. AC: Investigation, Writing−review and editing. MH: Funding acquisition, Resources, Supervision, Writing−review and editing. EB: Conceptualization, Data curation, Methodology, Project administration, Supervision, Validation, Visualization, Writing−original draft, Writing−review and editing.
