# Supplementary material for: Comprehensive ethological analysis of fear expression in rats using DeepLabCut and SimBA machine learning model
Source: Front Behav Neurosci. 2024 Aug 1;18:1440601. doi: 10.3389/fnbeh.2024.1440601 (PMC11324570; doi:10.3389/fnbeh.2024.1440601)
Supplement: Supplementary file 1 [file Data_Sheet_1.pdf]

Supplementary Material

1 Conditioned suppression of operant responding

1.1 Behavioral protocol

Each conditioned suppression experiment consisted of 4 main phases: training, fear conditioning, recovery period, and fear memory testing. In experiment 1, we assessed whether defensive behaviors during conditioned suppression were affected by shock intensity using both male and female rats. First, rats underwent daily training of self-administration for 0.2% saccharin (w/v; diluted in tap water; Sigma Aldrich, Germany) in 20-min sessions under a fixed ratio (FR) schedule of reinforcement, where a fixed number of active lever presses resulted in a 100ul-delivery of saccharin solution in a receptacle together with a 5s-cuelight indicating timeout period. Inactive lever presses had no consequences. Rats underwent training at least 5 days/week for 20 sessions. Once acquiring stable operant responding rate, rats were then habituated to a neutral tone (2.9kHz, 70dB, 2 min duration), presenting twice at the 3rd and the 9th minute of self-administration sessions for 4 sessions, to ensure that the tone itself did not suppress operant responses. Following training for operant responding and tone habituation (day1-24), rats were equally divided into 4 groups where each group underwent conditioning using either 0.4mA, 0.6mA, 0.8mA footshocks or no shock controls (n=8/group/sex) in chambers with a novel context without lever introduction (white light illumination with a wall with stripped pattern). After 5-min of initial habituation, rats received repeated 3 conditioning trials of 30s-tone (conditioned stimulus, CS, 2.9kHz, 70dB) co-terminating with 2s-footshock (unconditioned stimulus, US, 0, 0.4, 0.6, or 0.8 mA) with 3 min intertrial interval. After fear acquisition, rats underwent 3 sessions of saccharin self-administration (day26-28) to allow recovery of baseline operant responding rates. On the next day (day29), expression of conditioned fear was tested. For this, the CS was presented twice, with a duration of 2 min, starting at the 3rd and 9th minute of the self-administration session in the absence of the footshock US. Videos from fear testing were manually scored and used for training and validating DeepLabCut + SimBA workflow.

In experiment 2, we investigated the effect of diazepam (0.3 and 1 mg/kg) on the underlying defensive behaviors of conditioned suppression using the same protocol as experiment 1 unless stated otherwise. Male and female rats underwent training for operant responding (day 1-20) with habituation to the tone

on last 6 training sessions (day 21-26). Rats then underwent fear conditioning using either 0.4 mA or 0.8mA (3 trials, 30s CS, 2s US, 3 min intertrial interval; day 27). During recovery period, rats were habituated to intraperitoneal injection at least twice before fear testing (day28-31). Once baseline operant responding rates were stable, rats from each conditioning group were further divided into three subgroups: vehicle control (saline), diazepam 0.3 mg/kg and diazepam 1 mg/kg (n=10-18/subgroup). On the next day (day 32), rats received an intraperitoneal injection of either diazepam (0.3 or 1.0 mg/kg diluted in sterile water with 2% ethanol from diazepam 5 mg/ml, Richter Pharma AG, Austria) or saline 15 min before fear testing. Conditioned suppression was then assessed in fear testing and videos were used for training and validating DeepLabCut + SimBA workflow.

1.2 Calculation of the suppression ratio

Conditioned suppression was indexed by a suppression ratio. The rate of lever presses during CS (LP_{CS}) and during the 2-min baseline prior to the CS ($LP_{baseline}$) were recorded during each self-administration session with CS. To normalize the suppression of lever responding during CS with baseline operant responding rate, a suppression ratio was calculated as follows (Armony et al., 1997):

$$\text{Suppression ratio} = (LP_{CS} – LP_{baseline}) / (LP_{CS}+ LP_{baseline}).$$

Negative and positive suppression ratio indicates CS-induced suppression and facilitation of operant responses, respectively, whereas suppression ratio of 0 indicate no change of operant responding rate during CS. Subjects with low baseline operant responding rate ($LP_{baseline} < 5$/min) were excluded from analyses.

1.3 Results: conditioned suppression of operant responding is intensity-dependent

In experiment 1, following operant self-administration training for saccharin, rats underwent fear conditioning in a novel context without lever introduction using either 0, 0.4, 0.6, or 0.8 mA. Rats then underwent daily self-administration until baseline responding rate recovered. Conditioned suppression upon CS presentation was assessed in the fear testing session under self-administration condition. In male rats, we found that the degree of CS-induced suppression of operant responding was dependent on the intensity of conditioning footshock in male rats (**Supplementary Figure 1A**). Kruskal-Wallis ANOVA test showed a significant difference in the suppression ratio between the different shock intensities (χ^2 =19.72; $p<0.001$; df=3) with an inverse relationship between sum of rank suppression ratio and shock intensity (206 for 0.0mA; 132 for 0.4mA; 67 for 0.6mA and 59 for 0.8mA). Post hoc

analysis (Dunn's test) indicated a significant decrease in the suppression ratio compared to the control group (0.0mA) when the tone was paired with 0.6 and 0.8mA (p=0.002 and p=0.001, respectively; **Supplementary Figure 1B**), but not with 0.4mA (p=0.214).

Similar findings were observed in female rats where the degree of the conditioned suppression was also dependent on the intensity of the footshock during fear conditioning (**Supplementary Figure 1C**). Kruskal-Wallis ANOVA test showed a significance difference in suppression ratio between shock intensities (χ^2 =15.98; p=0.001; df=3). Shock intensity was inversely correlated with sum of rank suppression ratio in each group (159 for 0.0mA; 111 for 0.4mA; 53 for 0.6mA and 54 for 0.8mA). The Dunn's test indicated that the suppression ratio of 0.6 mA and 0.8 mA conditioning group were significantly decreased in compared to those of 0.0 mA group (p=0.011 and p=0.002, respectively) (**Supplementary Figure 1D**).

2 Feature permutation importance of SimBA behavior classifiers

2.1 Calculating feature permutation importance in SimBA

While building random forest classifiers, SimBA used filtered tracking data to extract 221 features that were divided into 8 categories according to measurement metrics (**Supplementary Table 1**). To examine how the model used each feature to classify behavior, we used a built-in module in SimBA to calculate feature permutation importance (Nilsson et al., 2020). This value represents a reduction in accuracy performance as measured by F1 score when a given feature is excluded from the learning model. The greater permutation importance indicates the higher contribution a given feature has on the classifier. Since most of the contributed feature for each classifier has permutation importance of 0.01, feature permutation importance was analyzed by feature category. Relative feature importance by category was calculated from sum of permutation importances of each category normalized by total permutation importances of each classifier. The percentage of relative feature importances by categories were presented as explainability metrics.

2.2 Results: feature permutation importance of SimBA behavior classifiers

Relative permutation importances were calculated for behavior classifiers in our SimBA iteration 4, a model that was trained on videos from experiment 1-3, and iteration 5, a model that was trained on only videos from experiment 3. A similar pattern of feature permutation importances was found

between the classifiers for the same behavior in both iterations. Specifically, Euclidean distances between any two tracked points is the most important feature category contributing for the classification of operant responding, grooming, sniffing, rearing, free-air whisking, and head scanning (**Supplementary Figure 6**). Conversely, the freezing classifier was mainly contributed by movements of tracked points (59.4%). The contribution of features in movement category was increased for freezing detection by iteration 5 (84.5%) compared to those by iteration 4 (59.4%). This resulted from an increase permutation importance of features derived from nose movement of the freezing classifier in iteration 5. Notably, the operant classifier in iteration 4 was partly dependent on features derived from detection probabilities of the tracked points, which is likely due to occlusion of body parts when rats were engaging in operant responding. Collectively, our findings suggest that behavioral classifications using both SimBA iterations are based on similar extracted features.

3 A step-by-step guide for implementing DeepLabCut + SimBA workflow

Here is a summary of 7 key steps for implementing DeepLabCut + SimBA workflow to perform ethological analysis of fear expression. A summarized flowchart is shown in **Supplementary Figures 6**.

3.1 Video pre-processing (Step 1)

Cut recorded videos into short videos containing only the period of interest (i.e., 30-120s length during the conditioned stimuli (CS) presentation in our experiment) using FFmpeg application.

3.2 Ethological analysis by manual scoring in Ethovision XT software (Step 2)

- Create an operational definition of each observable behavior.
- Score each behavior as a mutually exclusive start-stop event using a manual scoring function.
- Check time event plots to ensure that behavior did not overlap.
- Calculate percentage time spent on each behavior relative to the CS duration.
- Export manual scoring log files.
- For building classifier in SimBA, modify the log files to standardize annotated terms and correct file path using our custom-made python script (see in our shared repository: https://osf.io/yj7cb/?view_only=f01e4a3969ce46a28f41ca50683208c8.

3.3 Training neural network for creating pose estimation data in DeepLabCut (Step 3)

3.3.1 Create neural network training dataset

- Label videos. An eight-point labelling system (i.e., ear left, ear right, nose, center, lateral left, lateral right, tail base, tail end) was used to match the required inputs for subsequent post-processing step in SimBA.
- The default 95% of labeled frames were used to train ResNet-50 network, while the remaining 5% were used as a test dataset for neural network evaluation.

3.3.2 Train neural network and evaluate the network performance

- Optimize the number of training iterations, shuffles, and batch size by checking training loss, training and testing errors.
- The neural network improves its performance as the number of training iterations increases, reaching a plateau of maximum performance as indicated by training loss at approximately 500,000 training iterations under our setting.

3.3.3 Retrain the neural network if the performance and error is unsatisfactory by adding more training videos and extracting outlier frames and refining the labels.

3.4 Creating pose estimation data in DeepLabCut (Step 4)

- After achieving satisfactory network performance, the trained neural network in DeepLabCut can be used to create filtered tracking data for each video.
- The filtered tracking data was exported as CSV files.

3.5 Creating a SimBA project (Step 5)

- Import videos and filtered tracking data.
- Calibrate the distances in the videos into pixels per millimeter and perform outlier correction.
- Extract features in each video.
- Label behaviors by importing the modified scoring log files from Ethovision

- Designate videos to be training dataset and holdout videos for model validation. The number of training and validation dataset in our experiment were shown in **Table 1**.

3.6 Training machine learning model in SimBA (Step 6)

3.6.1 Build behavioral classifiers using a random forest model.

- The model was computed with the following hyperparameters: 600-2000 random forest estimators, 1-2 minimum sample leaf node, RF_criterion = gini, RF_max_features = sqrt, and test size = 20%. We found that a higher number of estimators is excessive for a relatively small training dataset. We decided to use a value of 500-1000 estimators to prevent overfitting.
- We acknowledged an imbalance of behavior representation in our training data; however, applying oversampling/undersampling in the parameter worsened the performance of machine learning prediction during pilot experimentation. Therefore, no sampling adjustment was set.

3.6.2 Assess accuracy performance and explainability metrics of the model

- With the analysis of 20% test frames from the training dataset, the accuracy performance for each classifier was measured in SimBA, reported as F1 score.
- Identify discrimination thresholds at F1 maximum (d_{F1max}) using precision-recall curve.
- Feature permutation importance can be calculated during the model training.

3.7 Validating the model using holdout videos (Step 7)

3.7.1 Identify adjusted discrimination thresholds (d_{adj}).

- An experimenter was examining 2 samples of validating videos that contained each behavior alongside the corresponding probability-time graphs to provide better classification of validation dataset.
- For classifiers of underrepresented behaviors, we noticed a greater variation among d_{adj} during the first manual inspection. Therefore, additional 2-3 videos were later examined to calculate mean adjusted discrimination threshold for underrepresented behaviors.

3.7.2 Create machine learning prediction using SimBA classifiers

- Classifiers were assigned with both d_{F1max} and d_{adj} before machine learning prediction.

3.7.3 Perform mutual exclusivity correction

- This step prevents any two behaviors from being classified as present at the same time.

3.7.4 Retrieve machine prediction results

- Calculate percentage time spent on each behavior relative to the CS duration similar to data obtained from manual scoring.

3.7.5 Determine inter-method reliability

- Calculate Pearson's r between data from machine learning prediction and data from manual scoring.
- If the machine prediction results are unsatisfactory, train new SimBA models by adding more training videos together with optimizing random forest estimators and undersampling/oversampling parameters.

4 References for information in supplementary materials

Armony, J.L., Servan-Schreiber, D., Romanski, L.M., Cohen, J.D., and LeDoux, J.E. (1997). Stimulus generalization of fear responses: effects of auditory cortex lesions in a computational model and in rats. *Cereb Cortex* 7(2)**,** 157-165. doi: 10.1093/cercor/7.2.157.

Nilsson, S.R.O., Goodwin, N.L., Choong, J.J., Hwang, S., Wright, H.R., Norville, Z.C., et al. (2020). Simple Behavioral Analysis (SimBA) – an open source toolkit for computer classification of complex social behaviors in experimental animals. *bioRxiv***,** 2020.2004.2019.049452. doi: 10.1101/2020.04.19.049452.

5 Supplementary Figures and Tables

5.1 Supplementary Figures

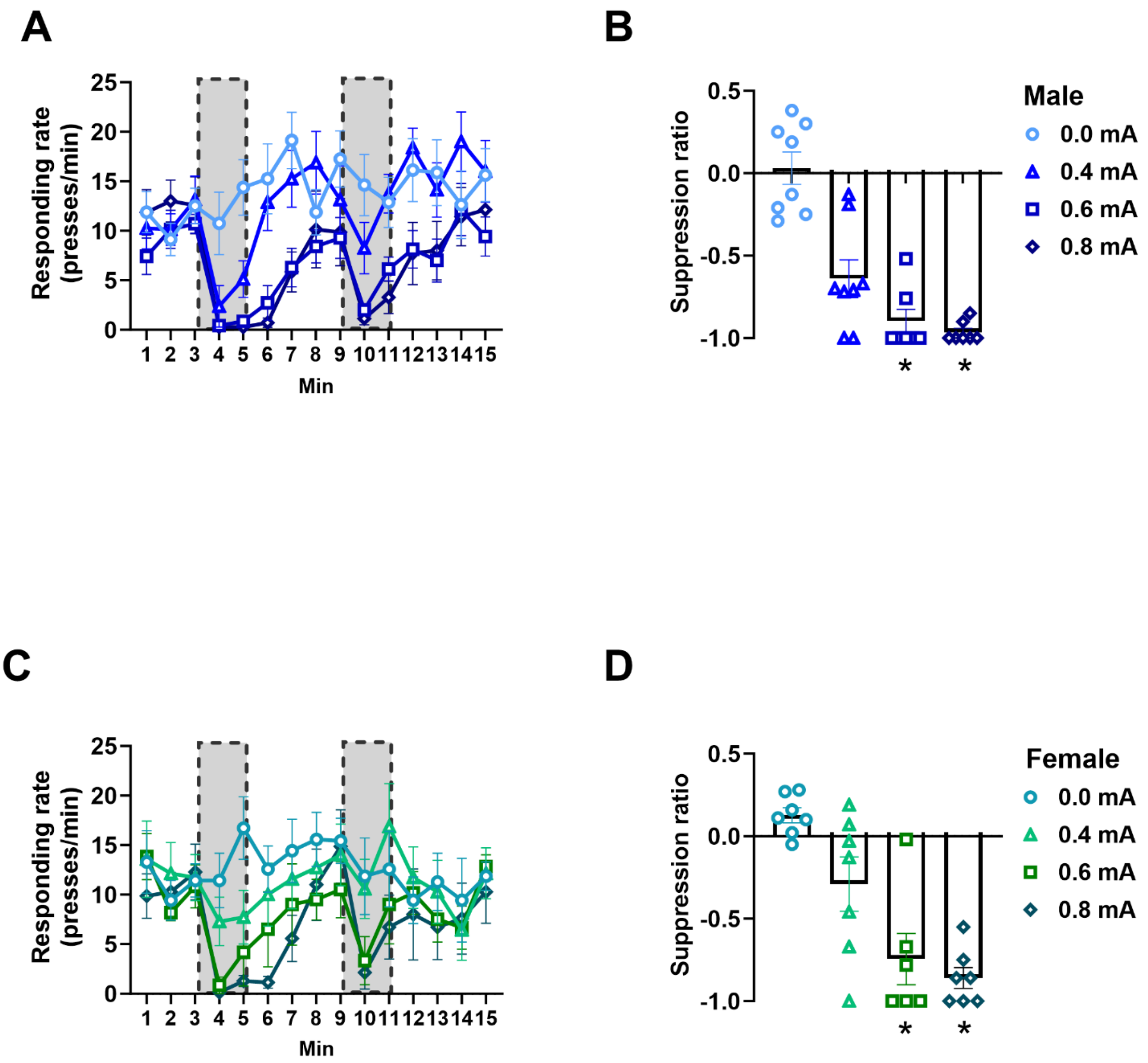

Supplementary Figure 1. Conditioned suppression of operant responding for saccharin varies as a function of shock intensity in both males **(A, B)** and females **(C, D)**. **(A)** Operant responding rate of male rats during fear testing. **(B)** Suppression ratio in fear testing of male rats that underwent conditioning with different footshock intensity. **(C)** Operant responding rate of female rats during fear testing. **(D)** Suppression ratio during fear testing of female rats that underwent conditioning with different footshock intensity. Grey boxes in **(A)** and **(C)** indicate the presence of the CS. Circles show data from 0.0 mA group. Triangles show data from 0.4 mA group. Squares show data from 0.6 mA group. Diamonds show data from 0.8 mA group. CS: conditioned stimuli. *, $p < 0.05$ compared between different conditioning footshock groups. Data are present as mean ± SEM.

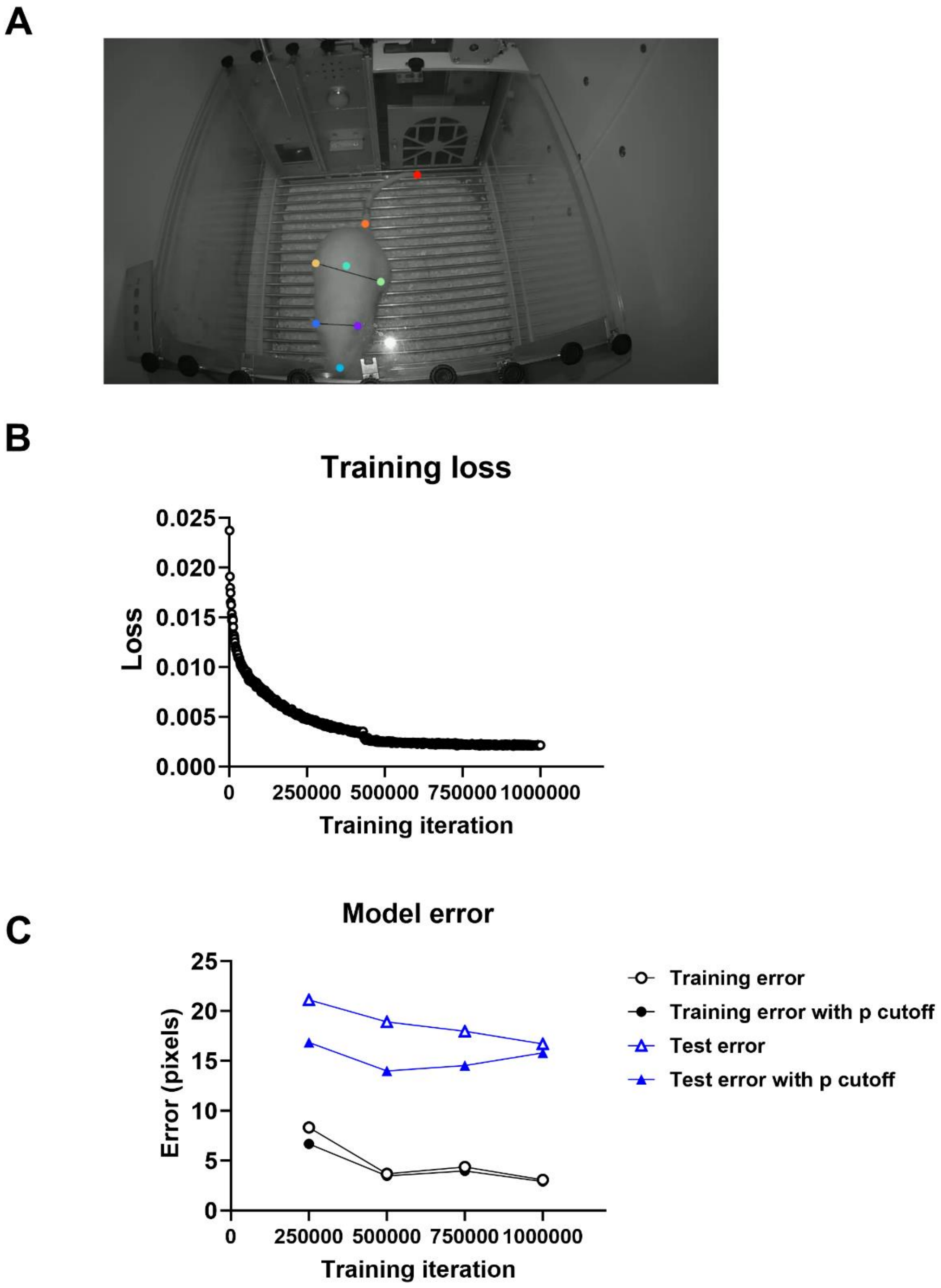

Supplementary Figure 2. Pose estimation and neural network training using DeepLabCut. **(A)** A representative image showing labeled body points in DeepLabCut. Pose estimation neural network training statistics **(B, C)**. **(B)** A model performance was indicated by training loss, which was decreased with an increase in training iterations. 500,000 training iterations were selected since training loss reached a plateau of maximum performance. **(C)** Model error in pixels was plotted for training and test dataset, both with and without a probability cutoff of 0.6 during the neural network training.

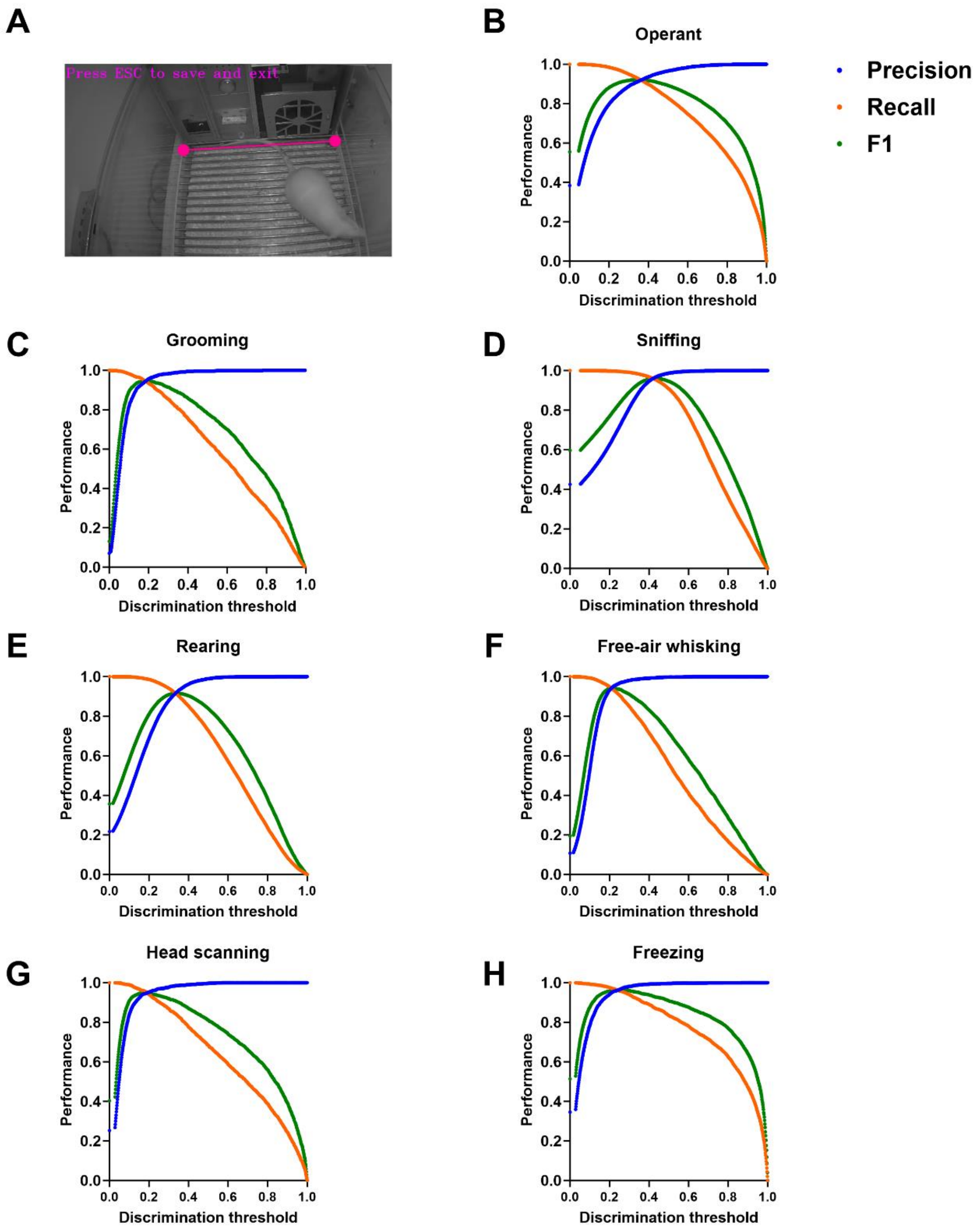

Supplementary Figure 3. Optimized parameters for building behavior classifiers in SimBA. **(A)** Calibration setting. The width of the operant chamber at the furthest distance from the camera was used as a reference for determining pixels per mm in the videos. Pixels per mm was then used during feature extraction to account for differences in frame resolution and distance from the camera to the operant chamber. **(B-H)** Precision-recall-F1 curves for determining discrimination threshold at maximum F1 for each behavior classifier in iteration 4, the iteration with the highest number of training dataset.

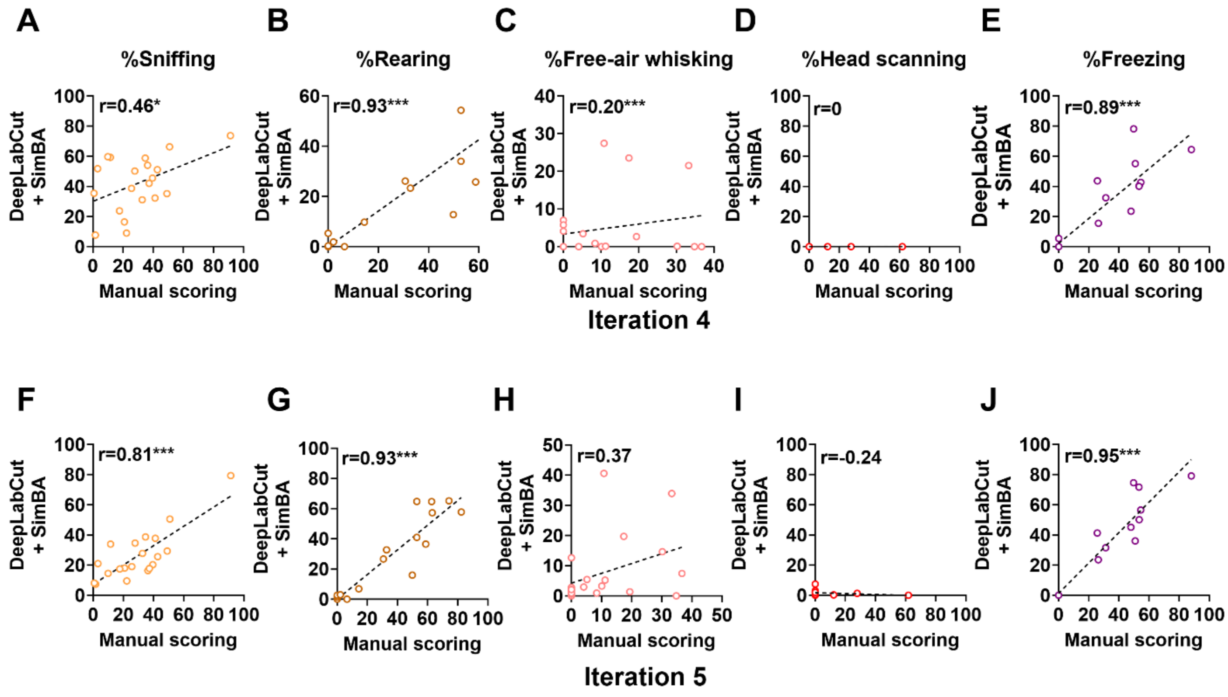

Supplementary Figure 4. Comparison of scores from DeepLabCut + SimBA workflow with scores from manual scoring while evaluating holdout videos from experiment 3. Correlation analyses of data derived from classifier in iteration 4 **(A-E)**, and iteration 5 **(F-J)**. The axes represent the percentage of time spent on each behavior. Note that head scanning rarely occurred and grooming did not occur in the validation dataset. *, $p<0.05$; ***, $p < 0.001$ compared between annotation methods.

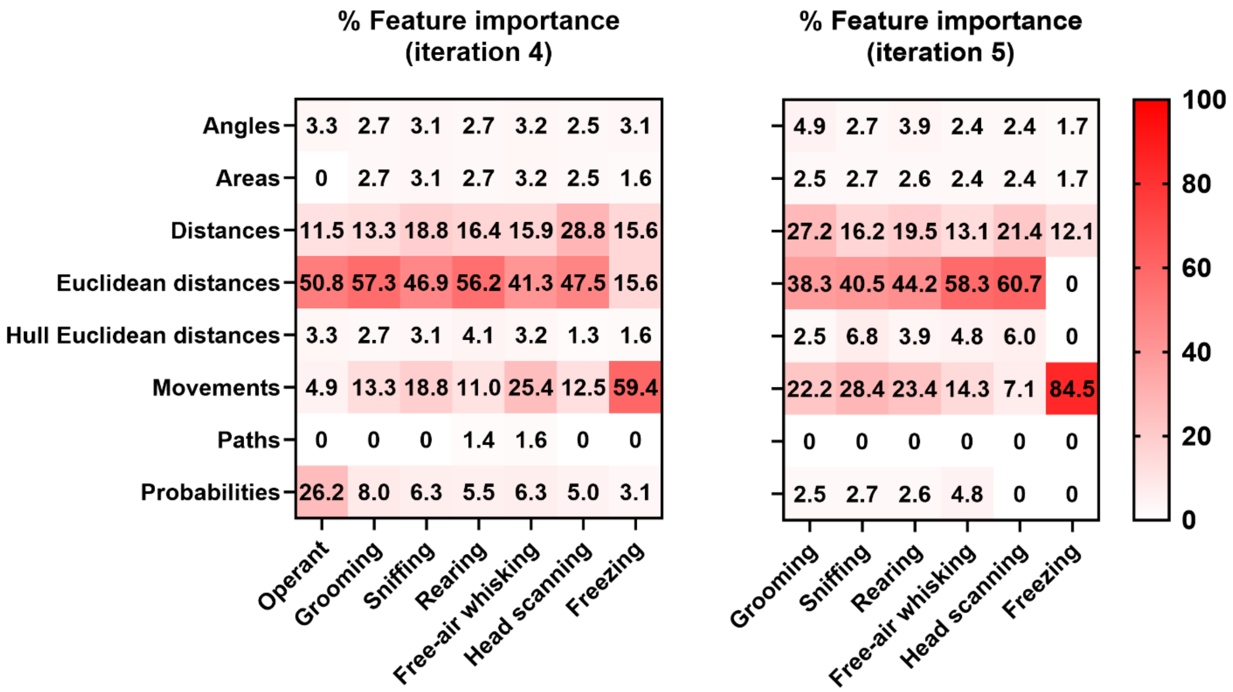

Supplementary Figure 5. Relative importance of feature categories as indicated by feature permutation importance. Sum of feature importance of classifiers from iteration 4 (left panel) and 5 (right panel). While training classifiers, SimBA calculated feature permutation importance which indicates the contributions from each extracted feature to behavioral classification by each classifier. Feature importances were summed by feature categories. Relative importance of each category was calculated by normalizing the sum of feature importances in the category to the total feature importance of each classifier. The numbers in the heatmaps are reported as percentages. The descriptions of each feature category are presented in **Supplementary Table 1**.

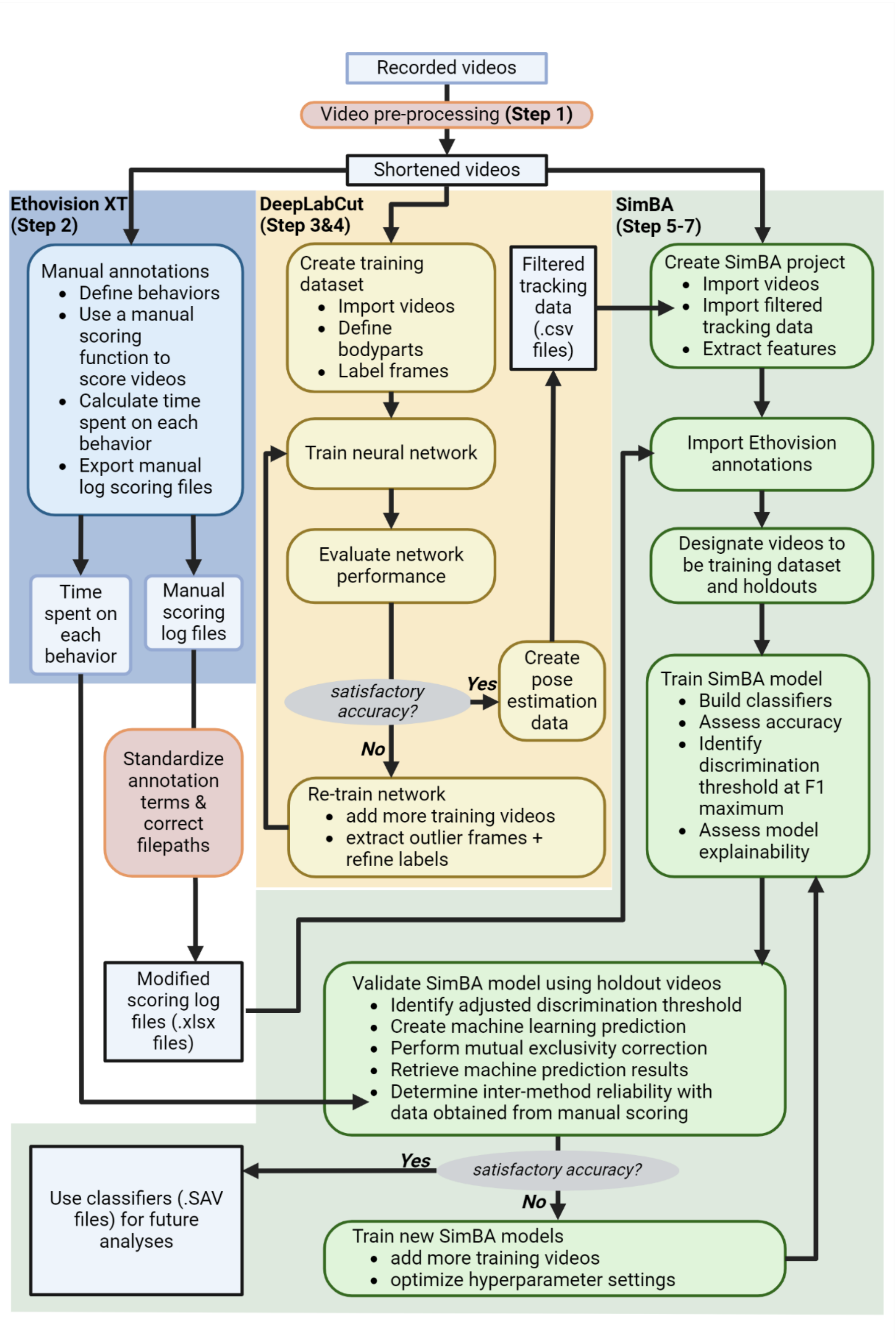

Supplementary Figure 6. Summary flowchart for implementing DeepLabCut + SimBA workflow to perform ethological analysis of fear expression. The figure was made using Biorender®.

5.2 Supplementary Table

Supplementary Table 1. Extracted features from pose estimation data for behavior classification in SimBA. All features can be divided into 8 categories based on measurement metrics.

Feature Category	Descriptions	Number of Features	Features using summary statistics
Angles	Body angle in degrees, calculating from the alignment of nose, centroid, tail base	2	Absolute value: 1, Standard deviation: 1
Areas	Area within the perimeter of a convex hull; change in area from the previous frame	3	Absolute value: 2, Standard deviation: 1
Distances	Distances between two tracked body points in each frame; aggregate functions of width distances over consecutive frames at 66ms, 133ms, 166ms, 200ms, and 500ms	23	Absolute value: 8, Mean: 5, Median: 5, Sum: 5
Euclidean distances	Aggregate functions of mean, smallest, and largest distance in straight line between any two tracked body points over consecutive frames at 66ms, 133ms, 166ms, 200ms, and 500ms	75	Mean: 15, Median: 15, Sum: 15, Standard deviation: 15, Percentile rank: 15
Hull euclidean distances	Mean, smallest, and largest distance in straight line between two tracked body points within the convex hull in the frame	7	Absolute value: 4, Standard deviation: 3
Movements	Movements of tracked body points from the previous frame; aggregation function of movement over consecutive frames at 66ms, 133ms, 166ms, 200ms, and 500ms	99	Absolute value: 15, Mean: 25, Median: 25, Sum: 25, Standard deviation: 2, Percentile rank: 7
Paths	Tortuosity of centroid trajectory over consecutive frames at 66ms, 133ms, 166ms, 200ms, and 500ms	5	Absolute value: 5
Probabilities	Sum of detection probabilities for all tracked body points in the frame; number of detections with detection probability lower than 0.1, 0.5, and 0.75	7	Absolute value: 4, Standard deviation: 1, Percentile rank: 2
